# Conserved untranslated regions of multipartite viruses: Natural markers of novel viral genomic components and tags of viral evolution

**DOI:** 10.1093/ve/veae004

**Published:** 2024-01-12

**Authors:** Song Zhang, Caixia Yang, Yuanjian Qiu, Ruiling Liao, Zhiyou Xuan, Fang Ren, Yafeng Dong, Xiaoying Xie, Yanhong Han, Di Wu, Pedro Luis Ramos-González, Juliana Freitas-Astúa, Huadong Yang, Changyong Zhou, Mengji Cao

**Affiliations:** Liaoning Key Laboratory of Urban Integrated Pest Management and Ecological Security, College of Life Science and Engineering, Shenyang University, 21 Huanan Street, Shenyang, Liaoning 110044, China; National Citrus Engineering and Technology Research Center, Integrative Science Center of Germplasm Creation in Western China (CHONGQING) Science City, Citrus Research Institute, Southwest University, 2 Tiansheng Road, Beibei, Chongqing 400712, China; National Citrus Engineering and Technology Research Center, Integrative Science Center of Germplasm Creation in Western China (CHONGQING) Science City, Citrus Research Institute, Southwest University, 2 Tiansheng Road, Beibei, Chongqing 400712, China; National Citrus Engineering and Technology Research Center, Integrative Science Center of Germplasm Creation in Western China (CHONGQING) Science City, Citrus Research Institute, Southwest University, 2 Tiansheng Road, Beibei, Chongqing 400712, China; Research Institute of Pomology, Chinese Academy of Agricultural Sciences, 98 Xinghainan Street, Xingcheng, Liaoning 125100, China; Research Institute of Pomology, Chinese Academy of Agricultural Sciences, 98 Xinghainan Street, Xingcheng, Liaoning 125100, China; Vector-borne Virus Research Center, College of Plant Protection, Fujian Agriculture and Forestry University, 15 Shangxiadian Road, Fuzhou, Fujian 350002, China; Vector-borne Virus Research Center, College of Plant Protection, Fujian Agriculture and Forestry University, 15 Shangxiadian Road, Fuzhou, Fujian 350002, China; College of Horticulture and Landscape Architecture, Southwest University, 2 Tiansheng Road, Beibei, Chongqing 400712, China; Laboratório de Biologia Molecular Aplicada, Instituto Biológico, Av. Cons. Rodrigues Alves 1252, São Paulo SP, 04014-002, Brazil; Laboratório de Biologia Molecular Aplicada, Instituto Biológico, Av. Cons. Rodrigues Alves 1252, São Paulo SP, 04014-002, Brazil; Embrapa Mandioca e Fruticultura, Rua da Embrapa, Caixa Postal 007, CEP, Cruz das Almas BA, 44380-000, Brazil; Hunan Agricultural University, 1 Nongda Road, Changsha, Hunan 410125, China; National Citrus Engineering and Technology Research Center, Integrative Science Center of Germplasm Creation in Western China (CHONGQING) Science City, Citrus Research Institute, Southwest University, 2 Tiansheng Road, Beibei, Chongqing 400712, China; Guangxi Citrus Breeding and Cultivation Technology Innovation Center, Guangxi Academy of Specialty Crops, 40 Putuo Road, Guilin, Guangxi 541010, China; Guangxi Key Laboratory of Germplasm Innovation and Utilization of Specialty Commercial Crops in North Guangxi, Guangxi Academy of Specialty Crops, 40 Putuo Road, Guilin, Guangxi 541010, China; National Citrus Engineering and Technology Research Center, Integrative Science Center of Germplasm Creation in Western China (CHONGQING) Science City, Citrus Research Institute, Southwest University, 2 Tiansheng Road, Beibei, Chongqing 400712, China

**Keywords:** multipartite viruses, untranslated regions, virus evolution, phylogenetics

## Abstract

Viruses with split genomes are classified as being either segmented or multipartite based on whether their genomic segments occur within a single virion or across different virions. Despite variations in number and sequence during evolution, the genomic segments of many viruses are conserved within the untranslated regions (UTRs). In this study, we present a methodology that combines RNA sequencing with iterative BLASTn of UTRs (https://github.com/qq371260/Iterative-blast-v.1.0) to identify new viral genomic segments. Some novel multipartite-like viruses related to the phylum *Kitrinoviricota* were annotated using sequencing data from field plant samples and public databases. We identified potentially plant-infecting jingmen-related viruses (order *Amarillovirales*) and jivi-related viruses (order *Martellivirales*) with at least six genomic components. The number of RNA molecules associated with a genome of the novel viruses in the families *Closteroviridae, Kitaviridae*, and *Virgaviridae* within the order *Martellivirales* reached five. Several of these viruses seem to represent new taxa at the subgenus, genus, and family levels. The diversity of novel genomic components and the multiple duplication of proteins or protein domains within single or multiple genomic components emphasize the evolutionary roles of genetic recombination (horizontal gene transfer), reassortment, and deletion. The relatively conserved UTRs at the genome level might explain the relationships between monopartite and multipartite viruses, as well as how subviral agents such as defective RNAs and satellite viruses can coexist with their helper viruses.

## Introduction

Regardless of viral host range, genetic material (DNA or RNA), genomic structure (circular or linear), genome polarity (plus, minus, or both), or gene arrangement, a virus genome is constituted by either one nucleic acid molecule, referred to as monopartite, or by multiple, relatively independent parts (Sicard et al. 2016). These parts can be segmented when assembled into a single viral particle or multipartite when individually packaged into physically separate virions (Sicard et al. 2016). The split genomes of segmented/multipartite viruses possess the capability of reassortment between genomic components of distinct viruses during mixed infections (Varsani et al. 2018), in addition to the genetic recombination shared with monopartite viral genomes (Simon-Loriere and Holmes 2011). Segmented/multipartite genomes are common among various genera or families across virus kingdoms, especially those that parasitize plants (Sicard et al. 2016; Lucía-Sanz and Manrubia 2017). However, it remains uncertain which came first in the evolutionary history of viruses: monopartite or segmented/multipartite viral genomes (Michalakis and Blanc 2020). When compared with monopartite genomes, segmented/multipartite genomes showcase certain advantages that have already been thoroughly summarized elsewhere (Lucía-Sanz and Manrubia 2017; Lucía-Sanz, Aguirre, and Manrubia et al. 2018; Michalakis and Blanc 2020), including better virion stability (Ojosnegros et al. 2011). These benefits provide a foundation for formulating a hypothesis regarding the evolutionary transition of viral genomes from monopartite to multipartite. This hypothesis remains plausible even if multipartite genomes arise through a potential mechanism of cheating in monopartite populations, making them comparatively less competitive (Leeks et al. 2023). Nevertheless, genomic segmentation also brings about movement costs within and between hosts, particularly for multipartite viruses (Michalakis and Blanc 2020). Despite this, a multicellular viral lifestyle that allows for spatial segregation and the possible delay of infection for certain genomic components in hosts might partly counteract these costs (Sicard et al. 2019).

*Kitrinoviricota* is a phylum under the realm *Riboviria*, with its positive-sense RNA viruses that do not infect prokaryotes grouped into a distinct cluster in the polymerase (Koonin et al. 2019). *Alsuviricetes* and *Flasuviricetes* are two classes in this phylum. The class *Flasuviricetes* includes a single family, the *Flaviviridae*, which was initially considered non-segmented until the discovery of jingmen-related species (Qin et al. 2014; Ladner et al. 2016). Another class, *Alsuviricetes*, comprises three orders, one of which is *Martellivirales*, where viral genome segmentation occurs frequently (https://viralzone.expasy.org/245). This order encompasses numerous members that infect plants, including the families *Closteroviridae, Kitaviridae*, and *Virgaviridae*. Additionally, there is a unique family, *Togaviridae*, exclusively infecting animals.

High-throughput sequencing (HTS) coupled with homology-dependent annotation (e.g. BLAST search) forms a classic combination that underpins viral metagenomic studies, enabling the dissection of constituents and structures of the virosphere (Roossinck, Martin, and Roumagnac et al. 2015; Shi et al. 2016, 2018). Homology found among conserved proteins shared by diverse viruses has significantly contributed to discovery of novel viruses, tracing the phylogenetic lineages and evolution, as well as the restructuring of the taxonomy (Koonin et al. 2020). In the meantime, certain protein-encoding open reading frames (ORFs) carried by viruses differ substantially from known sequences and are designated as ORFans, the diversity of which remains largely unexplored (Yin and Fischer 2008; Boratto et al. 2020). Identifying viral sequences where all ORFs are ORFans presents a challenge. Perhaps, the most effective approach for multipartite/segmented viruses is the search for conserved regulatory sequences within the untranslated regions (UTRs), by far (Li et al. 2015).

In this study, we designed an iterative BLASTn approach, powered by Python codes, that employs HTS contigs as queries and a regularly updated database of viral UTRs. We sequenced field plants to select HTS data containing multipartite-like viruses, subjecting the data to our UTR-based iterative BLASTn (UTR-iBLASTn) method. The resulting viral contigs were then subjected to online BLAST searches against NCBI databases to identify additional related viral sequences. The collaborative effort of computational analysis, field sampling, and online searching culminated in the discovery of numerous novel multipartite-like viral genomic segments. Each of the corresponding viruses features conserved genomic terminal sequences, and a substantial portion of these segments exhibit characteristics of small RNAs that are consistent with those found in plant viruses. The presence of equivalents in field plant samples and homologs within online datasets underscores their biological and evolutionary significance.

## Results

### Conserved UTRs of known multipartite/segmented viruses

**Figure 1.**
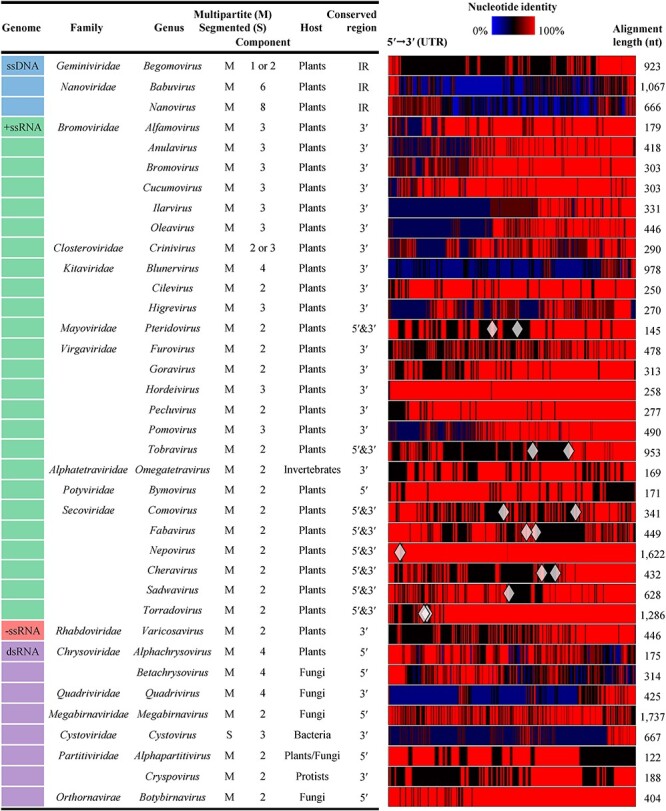
List of representative genera and families of multipartite/segmented viruses and multiple sequence alignment of conserved UTRs from split genomes of a selected type species (Supplementary Table S1) of each genus. The intact UTR sequence alignment for each species is shown as rectangular heatmap, with red indicating identical or highly identical regions. These representative genera were selected on account of that nucleotide (nt) identities calculated from the partial UTR sequence alignment (length ≥ 100 nt) generally exceeded 60 per cent (Supplementary Table S1). 5′ and 3′: the 5′ and 3′ genomic ends. 5′- and 3′-UTRs in the 5′&3′ alignments are separate with ◊ symbol. Some genera are considered as multipartite/segmented based on known phylogenetic relationships.

To identify conserved regions in split genomes, we analysed genomic sequences of multipartite/segmented viruses from over 91 genera spanning 33 families (Varsani et al. 2018). It was found that UTRs in viral genomes of many multipartite/segmented viruses remain conserved across different genomic components. Intergenic regions (IRs) within circular DNA genomes were considered as one type of viral UTRs. Linear genomes include both the 5′-UTR and 3′-UTR, situated at the respective genomic 5′ and 3′ termini. The left panel of Fig. 1 lists 37 representative viral genera (including one segmented genus) whose type species have genomes characterized by these conserved UTRs, along with 17 their corresponding family taxa. The detailed information for each type species (one per genus) is shown in Supplementary Table S1. Through sequence alignment of UTRs of different genomic segments for each species, conserved regions were identified, as depicted by the identity heatmaps in the right panel of Fig. 1. Generally, more than 60 per cent nucleotide (nt) identities were observed within conserved sequences (with an alignment length of at least 100 nt) in 5′-UTRs, 3′-UTRs, 5′- plus 3′-UTRs, or IRs for each species (Supplementary Table S1). It is worth noting that a majority of known multipartite viruses primarily infect plants, likely due to evolutionary advantages or statistical bias (Michalakis and Blanc 2020). Similarly, the hosts of the viral genera listed in Fig. 1 also demonstrate a strong bias towards plants.

### Design iterative BLASTx and UTR-based iterative BLASTn

**Figure 2.**
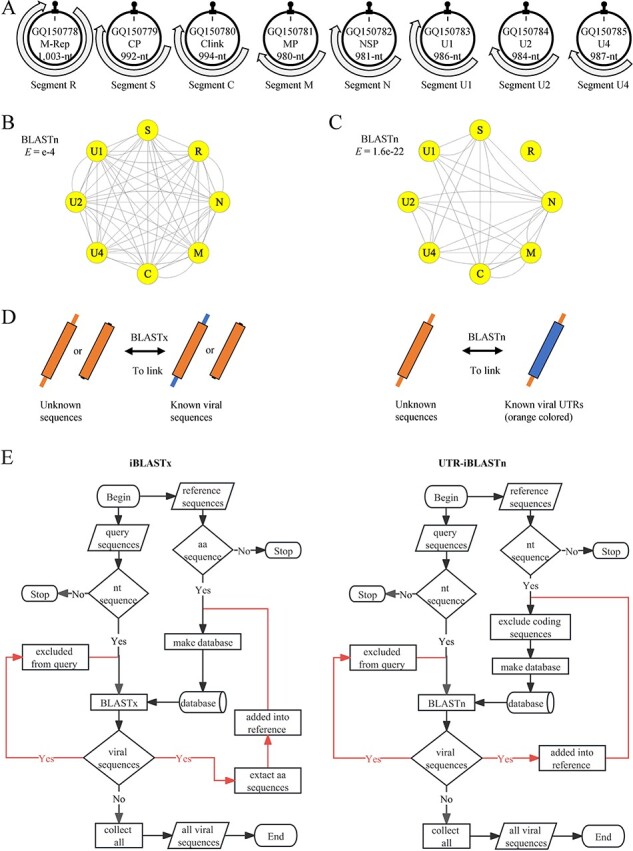
Demonstration of iterative BLASTx (iBLASTx) and UTR-based iterative BLASTn (UTR-iBLASTn). (A) The octapartite genome of FBNSV as an example, the IR of each genomic circular DNA is with a highly-conserved stem-loop (black panhandle). Networks based on reciprocal BLASTn relationships of FBNSV genomic segments are shown with a e-value cutoff of 1e-4 (B) and 1.6e-22 (C). (D) Situations where only BLASTx or BLASTn can be used; large rectangles represent proteins; same colors indicate sequence similarity. (E) Flowcharts for iBLASTx and UTR-iBLASTn.

Is it possible to utilize sequence conservation of viral UTRs to identify new viral genomic segments? Based on the concept illustrated in Fig. 1, if viral UTRs exhibit significant conservation, it becomes foreseeable that robust relationships between genomic components of a multipartite/segmented virus can be established through BLASTn of nucleotide sequences, independent of BLASTx that utilizes amino acid (aa) sequences. To demonstrate this, we employed the octapartite faba bean necrotic stunt virus (FBNSV, genus *Nanovirus*, GenBank accessions: GQ150778-GQ150785) as a practical example (Fig. 2A). By implementing a threshold e-value of 1e-4 (Fig. 2B), bidirectional relationships emerged between any two segments of FBNSV. Subsequently, we investigated the impact of different threshold e-values on the efficacy of UTR-based BLASTn for annotating genomic components. Notably, as the threshold e-value was moderately reduced to 1.6e-22, the R segment lost its connection with the other segments, resulting in a simplified relationship network (Fig. 2C). Therefore, setting an appropriate threshold e-value requires considering whether it is large enough to encompass more viral-like query sequences, yet small enough to exclude more non-viral queries. The criteria for different viruses and conditions are adaptable. Reciprocal BLASTn e-values (Supplementary Table S1), acquired for relationships between UTRs of each type virus species, are provided as reference criteria for those representative multipartite/segmented DNA/RNA viral taxa in Fig. 1. Nevertheless, it is important to note that viral contigs generated through HTS and subsequent sequence assembly might lack completeness in UTRs at the genomic ends. Thus, the actual BLASTn e-values for contigs of these representative viruses are likely to be larger than expected.

As shown in Fig. 2C, the U1 segment forms exclusive connections with the S, C, and N segments. These connections could serve as fundamental cues for pinpointing the U1 segment, assuming a hypothetical scenario in which BLASTn annotation involves both unknown and known segments. Assuming an iterative BLASTn strategy, one could initiate the process by identifying the segment M, followed by tracing the N segment through its association with M, and finally deducing the U1 segment through its connection with N. Consequently, adopting an iterative methodology becomes imperative when employing BLAST to explore unknown sequences, enabling more in-depth data mining.

There are two possible scenarios when utilizing BLAST tools to annotate unknown sequences using known viral sequences (Fig. 2D): (1) If the unknown sequences lack UTRs or conserved UTRs but possess conserved coding sequences, BLASTx should be employed. (2) If only the UTRs are conserved, BLASTn is the more suitable approach. In cases where both coding sequences and UTRs are conserved, either type of BLAST can be utilized. Building upon these observations, we developed the logic for an iterative BLASTx (iBLASTx) and an UTR-iBLASTn for annotating contigs derived from HTS. The primary steps in the flowchart for iBLASTx are as follows: (1) Input unknown HTS contigs as query sequences and any known viral sequences as references. (2) Create an aa sequence database using the reference sequences and initiate the first BLASTx. (3) Putative viral sequences newly identified by BLASTx are removed from the query sequences and translated (all ORFs that with length ≥ 150 nt) into aa sequences, which are added to reference sequences to expand the database. (4) Repeat BLASTx and the step (3) until no new viral sequences are predicted (Fig. 2E). For UTR-iBLASTn: (1) The references and the derived databases consist of any known viral UTRs. (2) Putative viral sequences newly annotated by BLASTn are removed from the query, and trimmed of coding sequences that exceed a user-defined threshold for aa length, and all the remaining untranslated sequences incorporated into the reference sequences for database expansion. (3) The iterative BLASTn process concludes when no new results are identified (Fig. 2E). Here, iBLASTx is considered a supplement method to UTR-iBLASTn. Both iBLASTx and UTR-iBLASTn were implemented based on NCBI-BLAST programs and Python code. We recommend manually alternating between iBLASTx and UTR-iBLASTn until both methods yield null results.

### Application of iBLASTx/UTR-iBLASTn method in sampled plants

To evaluate the performance of iBLASTx and UTR-iBLASTn tools in practical applications, fresh leaves of various plants were collected to generate HTS data. A single plant from each of the following species: ailanthus (*Ailanthus altissima*), apple (*Malus domestica*), camellia (*Camellia japonica*), citrus (*Citrus sinensis*), jasmine (*Jasminum sambac*), loquat (*Eriobotrya japonica*), and paper mulberry (*Broussonetia papyrifera*), was sampled from fields and subjected to sequencing, resulting in 8.38 to 14.33 gigabases (Gb) of clean reads. Data of each species were analysed separately and independently. The reads were subsequently assembled into contigs (Supplementary Table S2). Following DIAMOND-based local BLASTx and BLAST-based local iBLASTx analyses, multipartite-like viruses and a satellite-like subvirus with 27 contigs were predicted for the plant samples. These include two jingmen-related viruses (JMVs), one jivi-related virus (JVV), three bluner-related viruses (BNVs), two crini-related viruses (CNVs), and one crinivirus-associated satellite-related virus (Supplementary Tables S2, S3). Notably, the sequences of other putative viruses identified in these samples were not analysed in the scope of this study.

**Figure 3.**
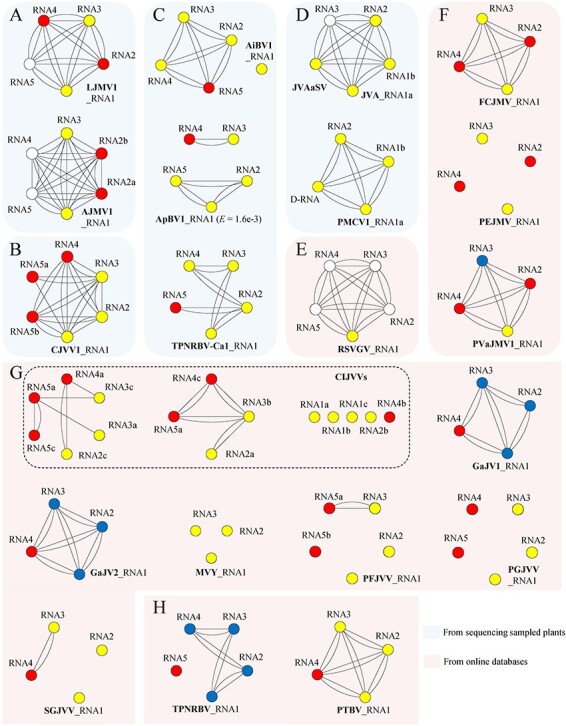
Homology relationships of genomic components within one or more related viruses that are constructed by BLASTx (colors of nodes) and UTR-BLASTn (lines between nodes) analyses of known or new multipartite-like RNA viruses newly annotated in this study. After BLASTx analyses, blue circles indicate known viral sequences from NCBI databases, yellow circles indicate new sequences related to known NCBI viral sequences, red circles indicate new sequences related to other new viral sequences, and white circles indicate new sequences unrelated to any known and new viral sequences. The lines between nodes indicate UTR-BLASTn e-value < 1.6e-3. (A) JMV (jingmen-related virus): LJMV1 (loquat jingmen-related virus 1), and AJMV1 (Ailanthus jingmen-related virus 1). (B) JVV (jivi-related virus): CJVV1 (citrus jivi-related virus 1). (C) BNV (bluner-related virus): AiBV1 (Ailanthus blunervirus 1), ApBV1 (apple blunervirus 1), and TPNRBV-Ca1 (tea plant necrotic ring blotch virus, isolate Ca1). E, e-value. (D) CNV (crini-related virus): JVA (jasmine virus A), and PMCV1 (paper mulberry crinivirus 1). JVAaSV, JVA associated satellite virus. (E) VGV (virga-related virus): RSVGV (Rhazya stricta-TSA virga-related virus). (F) JMV: FCJMV (Fagus crenata-TSA jingmen-related virus), PEJMV (Phalaenopsis equestris-TSA/SRA jingmen-related virus), and PVaJMV1 (Plasmopara viticola lesion associated Jingman-like virus 1). (G) JVV: CIJVVs (Carya illinoinensis-TSA jivi-related viruses, which are possible three coinfecting viruses), GaJV1/GaJV2 (grapevine associated jivivirus 1 and 2), MaVY (mastic virus Y), PFJVV (Pinus flexilis-TSA jivi-related virus), PGJVV (Picea glauca-TSA jivi-related virus), and SGJVV (Sarcandra glabra-TSA jivi-related virus). (H) BNV: TPNRBV, and PTBV (Paulownia tomentosa-TSA blunervirus).

Subsequently, UTR-iBLASTn was employed with a threshold e-value of 1e-2, leading to the identification of 14 additional putative novel viral contigs (Fig. 3A–D, Supplementary Table S2), which were not detected through iBLASTx analysis. This extended identification process expanded the potential count of genomic components associated with plant-related jingmenviruses (from 2 to 6) and jiviviruses (3 to 6), as well as the genera *Blunervirus* (4 to 5), and *Crinivirus* (3 to 4), when compared to the previously known genomic segments. These newly identified contigs, characterized by the presence of solely orphan genes (ORFans) and distinction from known viral sequences within databases, were provisionally labelled as orphan sequences (ORSs).

### Verification of viral ORSs by molecular cloning and online data searching

To substantiate the existence of newly identified putative viral segments or subviral agents, evidence was collected from various approaches. First, using viral-specific primers (Supplementary Table S4), the sequences of the identified contigs were validated through RT-PCR amplification. The amplified products were subsequently cloned and subjected to Sanger sequencing. In certain cases, the complete genomic segments of specific contigs were obtained by sequencing their terminal sequences. The verified 5′ and/or 3′ UTR sequences were conserved between different genomic segments of each virus. Importantly, this conservation remained consistent regardless of whether the genomic segments were complete or incomplete.

The obtained sequences of the 41 (27 plus 14) virus-like contigs were initially employed as queries to conduct further searches for homologous sequences using the online NCBI BLASTn and BLASTx tools. These searches utilized the virus-related (taxid:10,239) nr/nt and plant-related (taxid:3193) transcriptome shotgun assembly (TSA) databases. Additionally, certain viral sequence read archive (SRA) datasets (Supplementary Table S2) from NCBI, suspected to contain new sequences of known multipartite-like viruses, were downloaded and assembled into contigs for the same analyses as described earlier. Eventually, 55 previously roughly annotated or unannotated putative viral contigs or genomic sequences (including 44 from TSA, 8 from SRA, and 3 from GenBank) associated with 12 plant species were identified (Fig. 3E–H). These sequences potentially belong to 15 putative viral species (Supplementary Tables S2, S3). For instance, a contig of RNA1 of Plasmopara viticola lesion-associated Jingman-like virus 1 (PVaJMV1) had been identified previously. In this study, we reanalysed the corresponding sequencing data (accession no.: SRR11364892) available in the SRA database. As a result, a longer contig of 3,243 nt was obtained, surpassing the previous 3,173 nt (Supplementary Data S1, Table S3). Furthermore, while mastic virus Y (MVY) is a known entity, its three genomic segments stored in the GenBank database had not been previously identified as jivivirus-related (Fig. 3G). Among the 55 contigs or sequences, 30 were related to known viruses, 21 were ORSs that showed homology only to new sequences of the viruses identified from the seven sampled plant species, and four were ORSs that had no known homologs. The four ORSs and a TSA sequence (GAMW01005974.1) originating from *Rhazya stricta* among the 30 possibly belong to a novel virga-related virus (VGV) referred to as Rhazya stricta-TSA VGV (RSVGV). The remaining 14 viral species were classified into three groups: three were grouped as JMV, nine as JVV, and two as BNV (Supplementary Table S2).

### Field investigation of viral ORSs

To further support our findings above and to test persistence of the satellite virus and certain ORSs from the six viral species (with five being novel), the occurrence of these nucleic acid molecules in field plants was investigated by RT-PCR (Table 1). Seven or more plant individuals were tested per virus (including a helper virus). These plants had tested positive for each virus using RT-PCR and the primers for the corresponding replication-associated genomic segment. Proteins encoded by viral ORSs specific to plant hosts, rather than animals, can serve as RNA silencing suppressors, countering host defenses (Burgyán and Havelda 2011), and as movement-associated proteins, facilitating intercellular or systematic viral trafficking (Navarro, Sanchez-Navarro, and Pallas et al. 2019). However, none of these proteins was identified through homology analysis for all the JMV and JVV, and some BNV. This is likely attributed to significant sequence divergence from those found in known plant viruses. The detection rate of the ORSs and satellite ranged 20–100 per cent, highlighting the indispensability of some ORSs in viral infections (Table 1). In the case of the crinivirus originating from paper mulberry, tentatively named paper mulberry crinivirus 1 (PMCV1), a putative defective RNA (D-RNA) derived from RNA2 was occasionally detected (in 6 out of 17 samples). Remarkably, the homologous regions of RNA2 and the D-RNA differed by only 1 per cent in nucleotide sequence. The depth of coverage of paired reads across four specific deletion positions (eight nt positions) of the D-RNA exceeded 2,965. These observations suggest that the D-RNA may be replicable and/or producible within host cells, and/or transmissible between hosts.

**Table 1.** Detection of novel viral genomic components in plant host individuals positive for the corresponding genomic component encoding RNA-dependent RNA polymerase.

Virus	Infected plants	RNA	Detected plants	Detection rate (%)
LJMV1	12	RNA2	12	100
		RNA4	8	67
		RNA5	12	100
CJVV1	7	RNA4	7	100
		RNA5a	6	87
		RNA5b	7	100
ApBV1	22	RNA4	22	100
		RNA5	22	100
TPNRBV-Ca1	11	RNA5	11	100
JVA	15	RNA3	8	53
JVAaSV	15	RNA	3	20
PMCV1	17	D-RNA	6	35

Note: LJMV1, loquat jingmen-related virus 1; CJVV1, citrus jivi-related virus 1; ApBV1, apple blunervirus 1; TPNRBV-Ca1, tea plant necrotic ring blotch virus isolate Ca1; JVA, jasmine virus A; JVAaSV, JVA associated satellite virus; PMCV1, paper mulberry crinivirus 1.

D-RNA, defective RNA.

### Small RNA analysis of viral ORSs

**Figure 4.**
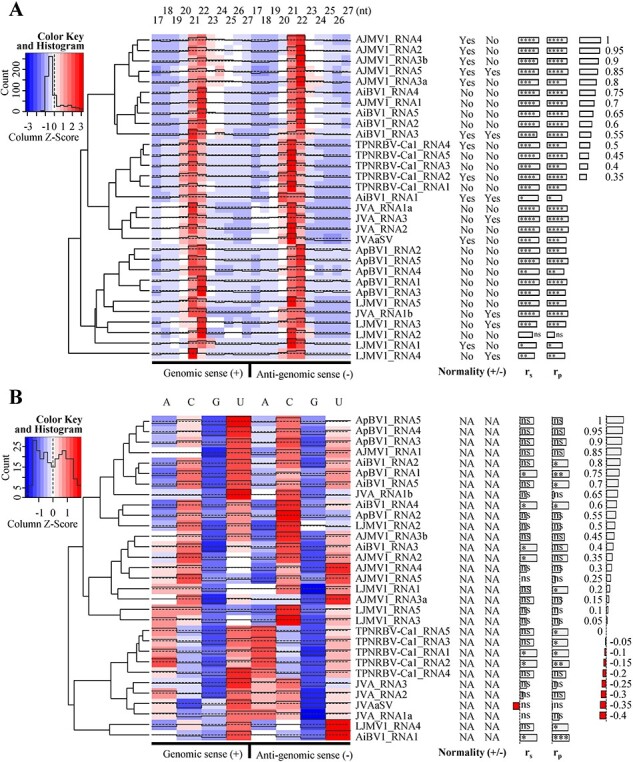
Cluster and heatmap analysis of distribution of virus-derived small interfering RNAs (vsiRNAs) in the size of 17-nt to 27-nt (A), and the 5′ nucleotide preference of A, C, G, and U (B), for certain viruses sequenced from sampled plants. The distributions of vsiRNAs from both genomic senses are concatenated from the genomic sense to the anti-genomic sense. Three-color gradients from blue to red indicate quantity from small to large of vsiRNAs distributed at a specific item. NA, not available. Normality, result of test by normal distribution. r_s_, Spearman correlation coefficient. r_p_, Pearson correlation coefficient. Symbols for *P* values of r_s_ and r_p_: ****, <1e-4; ***, 1e-4–1e-3; **, 1e-3–1e-2; *, 1e-2–5e-2; ns, >5e-2. Scale bars for values of r_s_ and r_p_ are placed in the rightmost position. AiBV1, Ailanthus blunervirus 1; AJMV1, Ailanthus jingmen-related virus 1; ApBV1, apple blunervirus 1; JVA, jasmine virus A; JVAaSV, jasmine virus A associated satellite virus; LJMV1, loquat jingmen-related virus 1; TPNRBV-Ca1, tea plant necrotic ring blotch virus, isolate Ca1.

To further support our findings above and understand association of different viral/subviral nucleic acid molecules, except citrus (1 JVV) and paper mulberry (1 CNV), other plant species infected by JMV, BNV, and CNV were sequenced to explore viral small RNA patterns, by referring to Aguiar et al. (2015). We focused on two aspects of the virus-derived small interfering RNAs (vsiRNAs): their size distribution within the range of 17–27-nt and the 5′ nucleotide preference (5′-nt) among A, C, G, and U. As the result, the vsiRNA size distributions across the viruses exhibited common peaks at 21-nt and 22-nt. This indicated that these viruses are significantly influenced by homologs of *Arabidopsis thaliana*’s Dicer-like (DCL) 4 and 2, according to both experimental and empirical data (Hull 2013). Clustering of viral sequences based on the size distribution or the 5′-nt preference of vsiRNAs seemed to be primarily influenced by variables such as the viruses and their respective hosts (Fig. 4A, B). The observed preference of the predominant 5′-nt for bases (A, C, U) other than G could be interpreted as an evolutionary inclination in virus-plant RNA silencing interactions (Fig. 4A). A significant and robust correlation (*P* value < 0.05 while correlation coefficient—r > 0.8) was frequently noticed between the genomic (+) and anti-genomic (-) senses of most viruses regarding the vsiRNAs size distribution. However, this correlation was less pronounced in connection with the preference for the 5′-nt (Fig. 4B). This aligns with the targeting specificity of DCLs for double-strand RNAs, contrasting with the recognition of single-strand RNAs by the host’s RNA-induced silencing complex through complementary small RNAs (Mi et al. 2008). In summary, it is reasonable to conclude that these viruses directly engage with the plant defense system at the intracellular level.

### Phylogeny of the orders Amarillovirales and Martellivirales

**Figure 5.**
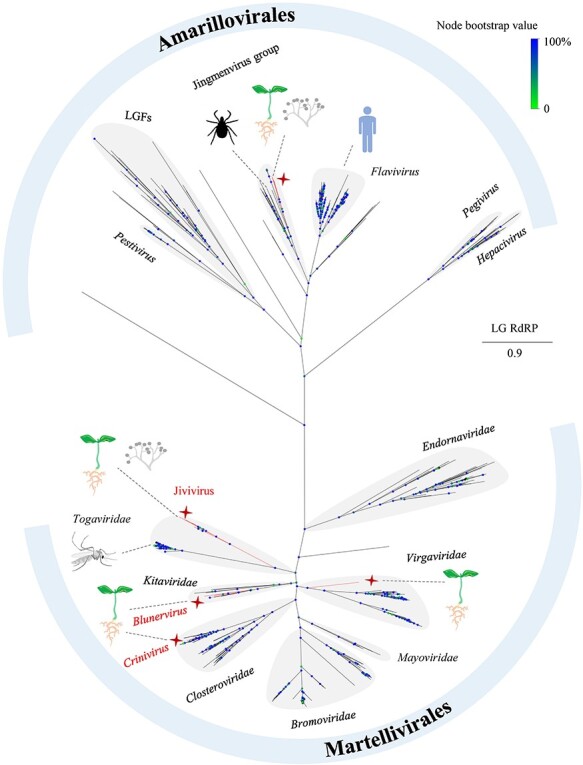
A phylogram inferred from amino acid sequence alignment of the RNA-dependent RNA polymerases (RdRPs) shows evolutionary positions of the new viruses or viral groups (marked in red) in the orders *Amarillovirales* and *Martellivirales*. LGFs: large genome flaviviruses. The splits at the nodes are supported by bootstrap analysis with 1,000 replicates. The scale bar represents the number of substitutions per site.

Preliminary analyses indicated that the newly discovered viruses are affiliated with the orders *Amarillovirales* and *Martellivirales* within the phylum *Kitrinoviricota*. However, their precise phylogenetic positions require further elucidation. Given the ubiquitous presence of RNA-dependent RNA polymerase (RdRP) in viruses in this phylum, a phylogenetic tree was constructed using RdRP amino acid sequences from the multipartite-like viruses annotated in this study and extant representative viruses in the phylum (Fig. 5). In this tree, the BNV and CNV were grouped into clusters corresponding to the genera *Blunervirus* and *Crinivirus*, respectively, indicating close relationships in both cases. In contrast, JMV formed a clade near the tick-infecting Jingmenvirus group; clades for JVV and VGV emerged distally within the families *Togaviridae* (animals as hosts) and *Virgaviridae* (plants as hosts), respectively; these signified distinctive evolutionary paths (Fig. 5). We compared these five types of viruses to their closest relatives at the protein level and observed that the aa sequence identities never exceeded 74.2 per cent.

### JMV and JVV

**Figure 6.**
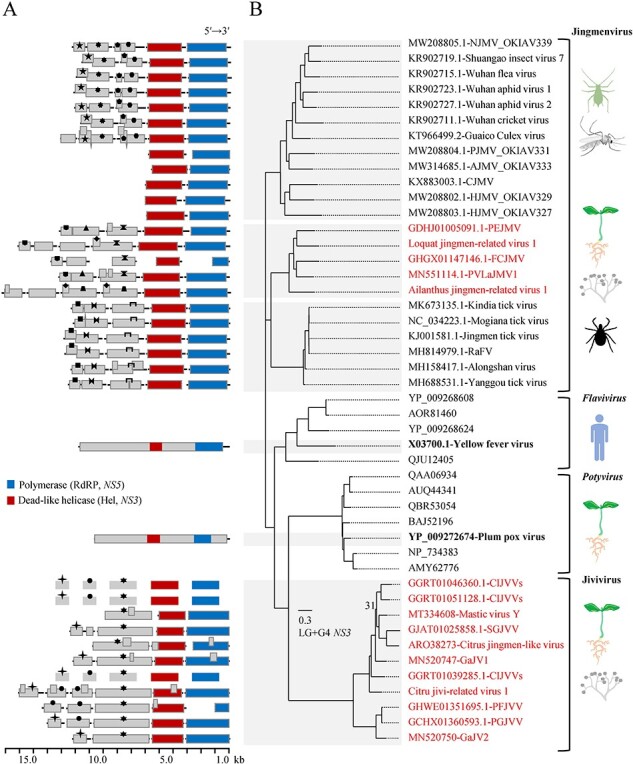
Genome sketch (A) and phylogenetic reconstruction with the LG + G4 model (B) of the representative viruses that are homologous in the *NS3* (DEAD-like helicase) gene according to the result of an online BLASTx search using viruses of the Jivivirus-group as queries. In the part A, the segmented horizontal lines, and the bars on the lines represent viral genomes, and the putative proteins possibly encoded, respectively; the blue, and red bars represent the RNA-dependent RNA polymerase (or *NS5* gene), and DEAD-like helicase (or *NS3* gene) domains/proteins, respectively; the gray bars with the same symbols indicate protein homologs; genome sketches of three CIJVVs (Carya illinoinensis-TSA jivi-related viruses) are equivalent due to possible coinfection of three highly-related viruses. In the part B, the tree is constructed from the *NS3* genes; the bootstrap value of a node is not shown unless it had less than 50 per cent value in the test with 1,000 replicates; the scale bar represents the number of substitutions per site; the accession no. of a viral genome is followed by its name, but only one typical virus of the genus *Flavivirus* or *Potyvirus* is detailed in name (in bold); the selected viruses for phylogeny can infect insects (e.g. aphid, mosquitoes), plants and/or fungi, ticks, and mammals (e.g. humans); the abbreviated virus names: NJMV, Neuropteran jingmen-related virus; PJMV, Psocopteran jingmen-related virus; AJMV, Arachnidan jingmen-related virus; CJMV, Changjiang jingmen-like virus; HJMV, Hemipteran jingmen-related virus; PEJMV, Phalaenopsis equestris-TSA/SRA jingmen-related virus; FCJMV, Fagus crenata-TSA Jingmen-related virus; PVLaJMV1, Plasmopara viticola lesion associated Jingman-like virus 1; RaFV, Rhipicephalus associated flavi-like virus; SGJVV, Sarcandra glabra-TSA jivi-related virus; GaJV1/GaJV2, grapevine associated jivivirus 1 and 2; PFJVV, Pinus flexilis-TSA jivi-related virus; PGJVV, Picea glauca-TSA jivi-related virus.

Considering the host and virus types, the sampled JMV and JVV were designated as Ailanthus jingmen-related virus 1 (AJMV1), loquat jingmen-related virus 1 (LJMV1), and citrus jivi-related virus 1 (CJVV1). Based on the data and database types, the JMV and JVV retrieved from NCBI were labelled as Fagus crenata-TSA jingmen-related virus (FCJMV), Phalaenopsis equestris-TSA/SRA jingmen-related virus (PEJMV), Carya illinoinensis-TSA jivi-related viruses (CIJVVs), Pinus flexilis-TSA jivi-related virus (PFJVV), Picea glauca-TSA jivi-related virus (PGJVV), and Sarcandra glabra-TSA jivi-related virus (SGJVV). PVaJMV1, grape associated jivivirus 1 and 2 (GaJV1, GaJV2), and MVY are previously known. FCJMV, PEJMV, and PVaJMV1 exhibited a genomic arrangement comparable to that found in animal-infecting jingmenviruses. This configuration is characterized by two core segments encoding NS5 (RdRP, RNA1) and NS3 (DEAD-like helicase superfamily, DEAD-like Hel, RNA3) proteins, along with two newly identified ORSs (RNA2, RNA4) likely coding for host-specific proteins (Fig. 6A). An additional ORS was found in genome of LJMV1. In the case of AJMV1, this ORS was absent, but two other ORSs appeared. For the sampled JMV, both the 5′ and 3′ UTRs are conserved in either case of the genome, complete (AJMV1) or not (LJMV1) (Supplementary Fig. S1). Regardless of the analysed NS5 and NS3 proteins, the phylogenetic trees consistently showed that the five plant/fungi JMV were clustered together with those infecting ticks, forming two parallel sub-clusters within the Jingmenvirus group (Fig. 6B, Supplementary Fig. S2).

The standard genome organization of jivi and jivi-related viruses is determined to be pentapartite, based on three known genomic RNA segments—RNA1 to RNA3. RNA1 codes for methyltransferase (Mtr) and helicase (Hel), RNA2 for RdRP, and RNA3 for DEAD-like Hel. The newly identified RNA4 and RNA5 each encode an unknown protein, speculated to be coat protein (CP) or movement protein (MP). There are two representative examples: CIJVVs (three coinfecting viruses) and PGJVV (Supplementary Fig. S3). Apart from the typical genome organization, genomic RNA duplication of RNA5, seemingly due to natural reassortment, was observed in CJVV1 and PFJVV. Another untypical scenario involves the absence of certain genomic RNA components in GaJV1 (RNA5), GaJV2 (RNA5), MVY (RNA 4 and 5), PFJVV (RNA4), and SGJVV (RNA5), which could be attributed to potential deficiency in the quality and/or annotation of the sequencing data. Conservation of sequence in the 5′ or 3′ UTRs of different genomic components is a hallmark observed in relatively complete viral genomes, such as CJVV1, GaJV1, and GaJV2 (Supplementary Fig. S3). Phylogenetic analysis using different protein domains (Mtr, Hel, and RdRP) does not alter the local topology of the formed trees. In these trees, the Jivivirus group of plant/fungus origins consistently branched alongside the mosquito-borne family *Togaviridae*, which exclusively infects animals (Supplementary Fig. S4A–C). However, in the tree for the *NS3* gene products (Fig. 6B), this group is most closely related to the monopartite families *Potyviridae* (the phylum *Pisuviricota*, infecting plants) and *Flaviviridae* (the phylum *Kitrinoviricota*, infecting animals). These findings contrast with previous suggestions that the closest affinity was to the Jingmenvirus group and family *Virgaviridae*. With the significant increase of sequencing data in the era of viromics, it is anticipated that new discoveries will help bridge the phylogenetic gaps among distantly related taxa, and this, in turn, will contribute to accurately positioning the evolutionary status of these emerging reassortant-like viruses.

### BNV, CNV, and VGVs

**Figure 7.**
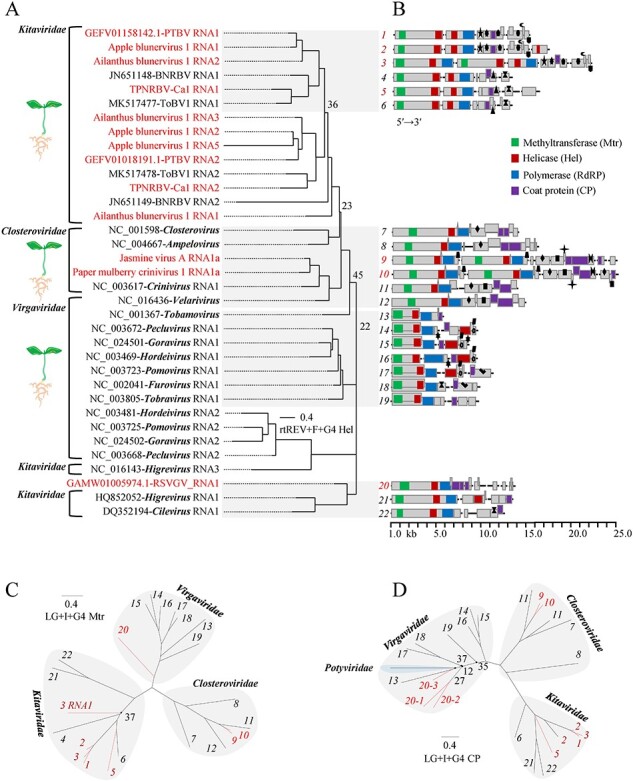
Phylogenetic analysis of the helicase (Hel) protein sequences of known representative and new plant-infecting, multipartite (-like) viruses in the families *Kitaviridae, Closteroviridae* and *Virgaviridae*, the order *Martellivirales* (A), and the corresponding genome sketch of each virus (B). Phylogenetic trees built based on viral methyltransferase—Mtr (C) and coat protein—CP (D). In the part A, the accession no. of a viral genome is before its name or the name of genus that the virus belongs to; the new viruses or viral isolate are indicated in red; the abbreviated virus names: PTBV, Paulownia tomentosa-TSA blunervirus; BNRBV, blueberry necrotic ring blotch virus; TPNRBV-Ca1, tea plant necrotic ring blotch virus isolate Ca1; ToBV1, tomato blunervirus 1; RSVGV, Rhazya stricta-TSA virga-related virus. In the part B, Mtr, RdRP, Hel, and CP are indicated by green, red, blue, and purple bars, respectively, and not defined regions indicated by gray bars; the protein homologs are signed identically. For all the trees, with bootstrap replicates of 1,000 adopted, only the bootstrap value of a node less than 50 per cent is shown; the scale bars, below which are best-fit models selected for tree construction, represent the number of substitutions per site; the same italic numbers in different trees represent the same viruses.

The BNV, CNV, and VGV were distinctively named based on the host, data origin, and virus attributes: Ailanthus blunervirus 1 (AiBV1), apple blunervirus 1 (ApBV1), tea plant necrotic ring blotch virus isolate Ca1 (TPNRBV-Ca1), Paulownia tomentosa-TSA blunervirus (PTBV), jasmine virus A (JVA, crinivirus), PMCV1, and RSVGV. The number of genomic components of previously identified members in the families *Kitaviridae, Closteroviridae*, and *Virgaviridae* is no more than four, three, and three, respectively. Like known blunerviruses, BNV have an RNA (RNA1 or AiBV1_RNA2) for Mtr and Hel, an RNA (RNA2 or AiBV1_RNA3) for Hel and RdRP, and an RNA (RNA3 or AiBV1_RNA4) for several proteins including the 24 K structural protein (SP24, CP) (Supplementary Fig. S5). AiBV1 (pentapartite), ApBV1 (pentapartite), and PTBV (quadripartite) share a novel genomic RNA encoding unknown proteins but lack an RNA for the 3a-like MP, suggesting a new genome combination type compared to the old one of TPNRBV and other blunerviruses. AiBV1 has a peculiar RNA1 containing Mtr, Hel, and RdRP domains of RNA1 plus RNA2 from known blunerviruses. It is analogous to RNA1s found in the genus *Cilevirus* with bipartite genomes or *Higrevirus* with tripartite genomes within the family *Kitaviridae*. AiBV1_RNA1 is phylogenetically closer to the genus *Blunervirus* than the two others in the three protein domains (Fig. 5, 7A). Thus, it can be designated as the prototype RNA1 of this genus, serving as an evolutionary intermediate of different genera. ApBV1 features a novel monocistronic RNA5 with a partial Hel domain, representing the third occurrence of this domain within the virus (Fig. 7B). TPNRBV-Ca1_RNA5 is inferred as a new ORS omitted previously, as an equivalent RNA was identified for the TPNRBV by reanalysis of the available original SRA dataset (SRR6459579). Each of these viruses is conserved in the genomic 3′ UTRs (Supplementary Fig. S5). Phylogenetic analysis based on Mtr, Hel, RdRP, and CP domains supported the differentiation of clustered bluner and BNVs into two groups: (1) AiBV1, ApBV1, and PTBV, and (2) the others (Fig. 5 and 7A, C, D). This observation suggests the potential existence of subgenera within the genus *Blunervirus*.

JVA and PMCV1 have two canonical genomic RNAs each: RNA1 for Mtr and RdRP; smaller RNA2 for two heat shock proteins (HSP 70 and 90), CP, and a minor CP (CPm); irrespective of other minor ORFs (Supplementary Fig. S5). A crini-related satellite named JVA associated satellite virus (JVAaSV) with its own CP was also discovered in association with JVA. Comparing JVA and PMCV1 with known criniviruses offers several differences: first, a duplicated RNA1 in both viruses; second, a putative persisting D-RNA derived from portions of PMCV1_RNA2; third, a novel ORS specific to JVA (RNA3). The signal of conservation among various RNAs associated with each of these viruses was primarily evident in the 3′ UTRs (Supplementary Fig. S5). In the context of phylogenetic analyses, the evidence provided by RdRP, Mtr, Hel, and CP domains collectively placed both viruses within the genus *Crinivirus* (Fig. 5 and 7A, C, D).

The family *Virgaviridae* typically consists of two or three genomic RNA segments that encode Mtr, Hel, RdRP, and a single CP, but RSVGV presents a new pentapartite genome organization, wherein Mtr, Hel, RdRP, and three CPs are integrated into RNA1, accompanied by the occurrence of four novel ORSs. It is important to note that the sequences from the TSA database have not yet been validated. The RNAs of RSVGV are highly identical in sequence of the 3′ UTRs (Supplementary Fig. S5). In the phylograms, while Hel showed the grouping with kitavirids (Fig. 7), Mtr, RdRP, and three CPs were associated with the family *Virgaviridae* (Fig. 5 and 7C, D).

### Gene repeats, segment duplication, horizontal gene transfer, and UTR phylogeny/networking

To gain deeper insights into the evolutionary historical processes of viruses, we conducted analyses on the conservation and collinearity, among segments of the same or different new multipartite viruses, of proteins and UTRs. First, multiple homologous proteins or proteic domains (aa sequence difference > 2.3 per cent) were identified in viral genomic components of 10 viruses, indicating potential evolutionary events such as speciation, tandem duplication, genetic reassortment, recombination, or deletion (Table 2). The extreme case (Supplementary Fig. S5), in terms of the protein repetition maximum three times, was found in RNA1–3 (one Hel domain for each) of AiBV1, in RNA1, 2, and 5 (one Hel domain for each) of ApBV1, and in RNA1 (three CPs) of RSVGV (if not chimeric). Three ORFs for RSVGV CPs and two ORFs for CP and CPm of PMCV1 and JVA are adjacent, pointing to gene tandem duplication events (Supplementary Fig. S6).

**Table 2.** List of multipartite-like viruses that possess two or three homologous proteins or protein domains.

Virus	Protein/domain	Frequency	RNA
AJMV1	unknown proteins	2	2a, 2b
CJVV1	unknown proteins	2	5a, 5b
PFJVV	unknown proteins	2	5a, 5b
AiBV1	Mtr	2	1, 2
	Hel	3	1, 2, 3
	RdRP	2	1, 3
ApBV1	Hel	3	1, 2, 5
TPNRBV-Ca1	Hel	2	1, 2
PTBV	Hel	2	1, 2
JVA	replication-associated proteins	2	1a, 1b
	CP (CPm)	2	2
PMCV1	replication-associated proteins	2	1a, 1b
	CP (CPm)	2	2
RSVGV	CP	3	1

Note: AJMV1, Ailanthus jingmen-related virus 1; CJVV1, citrus jivi-related virus 1; PFJVV, Pinus flexilis-TSA jivi-related virus; AiBV1, Ailanthus blunervirus 1; ApBV1, apple blunervirus 1; TPNRBV-Ca1, tea plant necrotic ring blotch virus isolate Ca1; PTBV, Paulownia tomentosa-TSA blunervirus; JVA, jasmine virus A; JVAaSV, JVA associated satellite virus; PMCV1, paper mulberry crinivirus 1; RSVGV, Rhazya stricta-TSA virga-related virus.

Mtr, methyltransferase; Hel, helicase; RdRP, RNA-dependent RNA polymerase; CP and CPm, coat protein and minor CP.

Three cases could be explained by segment duplication that relies on genetic mutation and reassortment: RNA1a and 1b of PMCV1 and JCA, and RNA5a and 5b of CJVV1. RNA duplicates (duplicate segment homologs) of each virus equal (PMCV1) or almost equal in length and are similar in ORF arrangement. RNA1s of PMCV1 and JCA, and RNA5s of CJVV1 shared 91.1 per cent and 67.8 per cent, and 59.8 per cent nt sequence identity, respectively.

In some cases, HGT (horizontal gene transfer) via recombination may occur after reassortment (Supplementary Table S3): RNA 5a and 5b of CJVV1 or PFJVV are homologous, but only RNA5a of each virus has a unique non-viral protein domain (CDD e-value < 1e-10). Recombination was only associated with one of the duplicate segment homologs, suggesting a random occurrence pattern of the HGT (Gilbert and Cordaux 2017).

**Figure 8.**
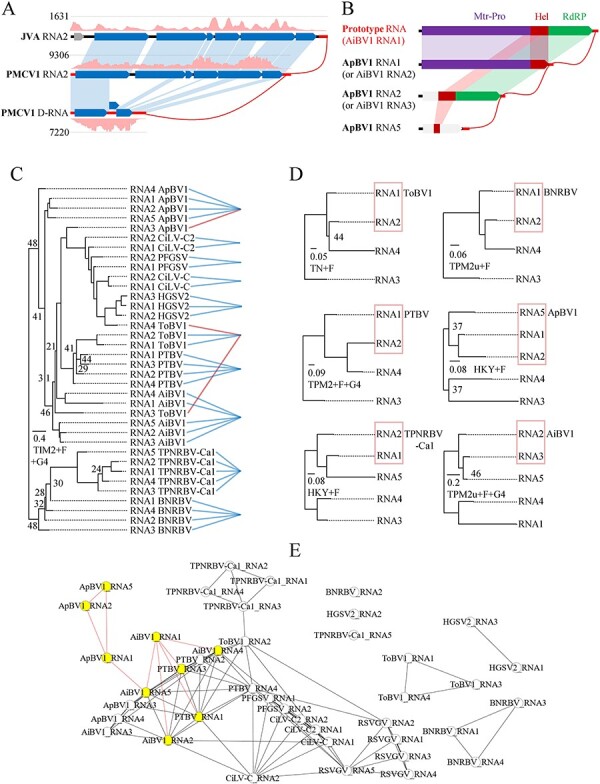
Trace of possible genetic deletion events of viruses in the genera *Crinivirus* and *Blunervirus*. Genetic deletion model involving RNAs of JVA—jasmine virus A and PMCV1—paper mulberry crinivirus 1 (A) or AiBV1—Ailanthus blunervirus 1 and ApBV1—apple blunervirus 1 (B). Detection of genetic deletion signal for the genus *Blunervirus* through 3′-UTRs phylogeny of related viruses (C) or each available blunervirus (D), since two different genomic RNA segments of blunerviruses share protein sequence homology (helicase). Networks constructed based on e-values (< 1e-4) of pairwise BLASTn between 3′-UTRs of genomic RNA segments of RSVGV (Rhazya stricta-TSA virga-related virus) and selected viruses in the family *Kitaviridae* (E). In the parts A or B, homologous regions are connected by bands in identical colors, and virus-derived RNA-seq reads mapped on genomic RNAs of JVA and PMCV1 (the highest read coverage number shown). In the parts C and D, as trees constructed with 1,000 bootstrap replicates, the bootstrap value shown at a node is less than 50 per cent; the scale bars represent the number of substitutions per site, and the selected tree models shown below. In the part E, close relationship between ApBV1_RNAs (1, 2, and 5) and between AiBV1_RNA1 and other typical blunerviral RNAs are highlighted by red lines and yellow nodes. The abbreviated virus names: BNRBV, blueberry necrotic ring blotch virus; PTBV, Paulownia tomentosa-TSA blunervirus; TPNRBV-Ca1, tea plant necrotic ring blotch virus isolate Ca1; ToBV1, tomato blunervirus 1; HGSV2, hibiscus green spot virus 2; CiLV-C, citrus leprosis virus C; CiLV-C2, citrus leprosis virus C2; PFGSV, passion fruit green spot virus.

Genetic deletion could result in partial sequences of the original viral RNA, as seen in putative D-RNA of PMCV1 from its RNA2, and possibly PMCV1_RNA2 from JCA_RNA2 (Fig. 8A). Interpreting RNA2 and 3 of AiBV1 and RNA1 and 2 of ApBV1, PTBV, and TPNRBV-Ca1 as outcomes of the same genetic deletion events (Fig. 8B) before speciation is reasonable. It is bolstered by the fact that Hel domains of the two types of RNAs showed similar viral phylogenetic topology and clustered in single clade (Fig. 7A), and the corresponding UTRs of each virus were phylogenetically closely related (Fig. 8C, D). In particular, three Hel domains of ApBV1 likely resulted from three independent deletion events: (1) RNA1 from deletion of a *Higrevirus*- or *Cilevirus*-related prototype RNA1 at the RdRP regions, (2) RNA2 from deletion of the Mtr regions in the prototype RNA1, and (3) RNA5 from ApBV1_RNA2 deleted at the RdRP domains. These were supported by phylogenetic analyses of the Hel domains (Fig. 7A) and 3′ UTRs (Fig. 8C, D), and by BLASTn-based networking of the 3′ UTRs (Fig. 8E), which consistently showed close relationships of the RNA1, 2, and 5. Intriguingly, AiBV1_RNA1, postulated to be a molecular relic of the blunerviral prototype RNA (Fig. 8B), is distant from (or not closely related to) the structural analogues of the two other genera, while closely related to the genus *Blunervirus* at both aa and nt levels, as shown in phylogenies of Mtr (Fig. 7C), Hel (Fig. 7A), and RdRP (Fig. 5) domains, and the 3′ UTRs (Fig. 8C), and in the 3′ UTR-network (Fig. 8E).

## Discussion

Based upon several earlier BLAST concepts (De La Peña and García‐Robles 2010; Li et al. 2015), we formulated the UTR-iBLASTn method to anchor the viral genomic components occupied by ORFans within data flows. It was employed to examine the data of many putative multipartite viruses belonging to two classes in the phylum *Kitrinoviricota*, leading to the discovery of many novel components, including the ORSs. Its application can potentially be expanded to encompass more groups of viruses from various hosts (Fig. 1), even monopartite species that have not been previously tested for ORSs, as exemplified by RSVGV. A crucial point of the method is that HTS and assembly generated viral contigs with UTRs being as full as possible to include core informative sites. Regarding this, the strategies below may be helpful to obtain satisfied HTS contigs: (1) choose symptomatic individuals for sampling, (2) deploy an appropriate sequencing method (e.g. long reads of 150 nt or longer, and rRNA-depleted RNA-seq for a broad spectrum of viral genomes and RNA derivatives), and (3) enlarge data size per sequencing.

The existence of flavi-related multipartite-like viruses possibly from plant/fungus hosts have been recognized previously but inadequate information impeded labelling and taxonomic clarity (Matsumura et al. 2017; Chiapello et al. 2020a, 2020b). Here, two JMVs from plant species sampled in fields exhibit unambiguous plant virus attributes in vsiRNAs (Fig. 4). However, it is still ambiguous whether hosts of the jivi-related viruses are plants, fungal endophytes, or even both (Silva et al. 2021). In the analyses of genomics and phylogenetics, the plant JMV clearly descended from a single origin with jingmenviruses infecting animals such as ticks (Fig. 6, Supplementary Fig. S2), but the jiviviruses and JVV likely had multiple origins including the viral families *Togaviridae* (RNA 1 and 2) (Fig. 5, Supplementary Fig. S4) and *Potyviridae* (RNA3) (Fig. 6), and fungus and bacterium (RNA 4 and 5) (Supplementary Table S3). Restricting the scope of phylogeny to the order *Martellivirales*, our newly discovered or annotated viruses may serve as evolutionary bridges between the families *Kitaviridae* and *Virgaviridae* (RSVGV) (Fig. 5, 7, 8) or genera of the former (AiBV1) (Fig. 7). Genetic reassortment, recombination, or deletion may be responsible for these intriguing evolutionary features.

Genetic mutations (accumulative substitutions, deletions, and insertions), recombination, and reassortment can lead to the emergence of new genomic segments in multipartite viruses. The persistence of existing segments might be challenged by the genome formula (GF), especially if these segments are low-frequency and dispensable (Sicard et al. 2013; Gallet et al. 2022). The shift in host and/or environment influences viral genetic mutation (resulting in specific quasi-species) (Schneider and Roossinck 2001), recombination (the HGT) (Irwin et al. 2022), reassortment (due to mixed infections) (Vijaykrishna et al. 2015), and the establishment of the setpoint GF (Zwart and Elena 2020; Gallet et al. 2022). Therefore, known multipartite viruses that undergo shifts between different hosts and/or environments (often facilitated by vectors), which in turn promote the acquisition and loss of genomic segments, warrant greater attention based on our findings.

Virus-encoded polymerase plays a crucial role in replication of multipartite viruses within hosts. The balance between the polymerase-encoding genomic segment and other segments fluctuates during viral infection due to the GF. Past studies have revealed that this particular segment accumulates at a relatively faster pace or to a greater extent in certain viruses (Wu et al. 2017; Zhao et al. 2019). Therefore, in this study, the presence of this segment was utilized as a prerequisite to assess the co-occurrence of other viral or subviral RNA molecules. The results indicated that the majority of the examined RNAs exhibited a close co-occurrence with their respective polymerase-encoding segments. However, a subset of the RNAs, including JVAaSV and PMCV1_D-RNA, did not exhibit this close association (Table 1). It is well established that satellites and D-RNAs, even though they may hold biological significance, do not consistently co-migrate with their associated helper viruses (Graves, Pogany, and Romero et al. 1996; Simon, Roossinck, and Havelda et al. 2004). Consequently, our method holds the potential for discerning whether a segment is essential (genomic segment) or not (satellite and D-RNA).

PMCV1_D-RNA seems replicable (Fig. 8A) and reproducible (Table 1) in host plants. Unlike defective interfering RNAs (DI-RNAs) that modulate symptoms of their helper viruses (Pathak and Nagy 2009), PMCV1_D-RNA appears not to affect the symptom severity at a macroscopic level. Long-lasting persistence of PMCV1_D-RNA is possibly supported by its complete UTRs, high copy number (Fig. 8A), and putative encapsidation with intact CPm that mediates between-host transmission via insects (Ng et al. 2021). DI-RNA (D-RNA) benefits or interferes with lifecycle of the corresponding helper viruses, shaping their evolution (Vignuzzi and López 2019; Leeks, West, and Ghoul et al. 2021), and can interact with other co-infecting subviral agents such as satellites (Qiu and Karen-Beth 2001). More investigation is needed to determine the potential role that putative D-RNA of PMCV1 plays.

It remains unclear if monopartite or segmented/multipartite form emerged first, each form possesses unique advantages and costs (Michalakis and Blanc 2020), and besides phylogenetic support on this issue is still scarce (Ramos-González et al. 2020). A stable complementary system consisting of multiple defective genomes could derive from monopartite viral genome through genetic deletion (Nee 1987; García-Arriaza et al. 2004; Lucía-Sanz and Manrubia 2017), implying monopartite as the possible earlier form. Three cases provided in our study make this implication more plausible: PMCV1, ApBV1, and AiBV1. It is likely that PMCV1_D-RNA has transitioned from transient to persistent by unknown mechanisms after its generation from regional deletions of the RNA2 (Fig. 8A, Table 1). The division of an original RNA through selective partial deletions could potentially elucidate the phenomenon of three Hel homologs originating from different yet closely related ApBV1 RNAs, all of which shared conserved UTRs (Fig. 7, 8). The presence of the original RNA was corroborated by the discovery of AiBV1 RNA1, which shares structural similarities with those found in the genera *Cilevirus* and *Higrevirus*; however, its phylogenetic placement leaned towards those in the genus *Blunervirus* (Fig. 7, 8). The relative conservation of UTRs in genomic segments of multipartite viruses renders them a valuable tool for studying the genetic deletion-based evolution of various viruses and segments.

The components in a multipartite genome appear to be complementary and organized. This rule can even cover viral parasites, the subviral satellites, from which their helper viruses may gain pathogenicity, and their relationship may become interdependent (Zhou 2013). Furthermore, viral UTRs contain a concentration of regulatory elements or signals for stability, transcription, replication, and packaging (Dreher 1999). In the cases of PMCV1_D-RNA and JVAaSV (Supplementary Fig. S5), their UTRs demonstrated homology with those of their respective helper viruses, which likely ensures compatibility. Similarly, other endogenous or exogenous nucleic acid molecules and their duplications with conserved UTRs might also become integrated into a viral genome during coevolution, either through cooperation or cheating (Leeks, West, and Ghoul et al. 2021). This could be the origin of some viral ORSs. Nevertheless, it remains perplexing that viruses with a fully functional genome system possess triple repetitions of protein domains such as Hel (Supplementary Fig. S5). Protein duplications may serve distinct functions, coordinate with each other, or even enhance the same function (Hughes 1994; Satyanarayana et al. 2004; Panchy, Lehti-Shiu, and Shiu et al. 2016).

In summary, using the UTR-iBLASTn method and our or open-sourced data, we identified new multipartite-like viruses with novel genomic components that fill evolutionary gaps or blanks and emerge as new phylogenetic twigs. These data deepened our understanding of viral genome segmentation and expanded our knowledge of virus diversity and their split genomes, particularly in variation of genomic segments.

## Materials and methods

### Analysis of segmented/multipartite viruses with conserved UTRs

Viral genera characterized by split genomes were sourced from the review by Varsani et al. (2018) and the Genome stats of ViralZone (https://viralzone.expasy.org/). A representative species from each genus was randomly chosen based on the virus taxonomy compiled by the ICTV (https://ictv.global/taxonomy; accessed as of 2020) to conduct an analysis of nucleotide sequence homology between UTRs of its different genomic components. UTRs were manually extracted using existing annotation data from GenBank format files downloaded from NCBI. Subsequently, UTRs of different genomic segments of each virus were aligned separately (Fig. 1), using the CLC Genomics Workbench 11 (Qiagen, Hilden, Germany). To enhance the alignment quality, less conserved regions (gaps occur in ≥ one-third of the segments that have been aligned, length > 5-nt) within the sequence alignment were manually removed, but it was ensured that the final alignment retained a minimum length of 100 nt (Supplementary Table S1). The nt identities were calculated from the final alignment, and if the identities surpassed a threshold of 60 per cent, the corresponding viral genus and family were included in Fig. 1. In total, at least 91 final alignments (one per virus per genus) were tested (Varsani et al. 2018), and 37 among them selected. To visually represent the degree of nt identity at each alignment position, a heatmap was generated from the intact sequence alignment of UTRs for each virus. This heatmap utilized blue-black-red color gradients to depict the range of identity, transitioning from low to high degrees of identity, and was produced using the CLC Genomics Workbench 11.

### Python-coded programs for iterative BLAST

The function of iterative BLAST was implemented using Python codes based on the BLAST programs from NCBI. Four distinct iBLAST tools were developed and made available in an online repertory (https://github.com/qq371260/Iterative-blast-v.1.0), including UTR-iBLASTn (Fig. 2), iBLASTx, iBLASTp, and iBLASTn/itBLASTx. It should be noted that iBLASTp and iBLASTn/itBLASTx were included in the repository although they were not utilized in this specific study. In iBLASTp, both the input query and database require protein sequences, while in iBLASTn/itBLASTx, nucleotide sequences are needed. In these analyses, the sequences inputted for database will be directly constructed into the database. Prior to running, it is essential to place all the tools into the ‘bin’ directory of the installed BLAST programs (tested version: 2.12.0+). The parameters used to execute BLAST are all applicable to iBLAST programs, allowing users to input single or multiple parameters in a blank using the same vocabulary and syntax. Notably, the default -outfmt parameter for intermediate temporary files is fixed at 6, and cannot be modified.

### Sample collection

Leaf samples exhibiting virus-like symptoms were collected from seven distinct plant species during comprehensive field studies conducted across three provinces in China: Chongqing (involving camellia—*Camellia japonica*, citrus—*Citrus sinensis*, loquat—*Eriobotrya japonica*, and paper mulberry—*Broussonetia papyrifera*), Liaoning (ailanthus—*Ailanthus altissima* and apple—*Malus domestica*), and Yunnan (jasmine—*Jasminum sambac*) (Supplementary Table S2). These collected samples served as the primary materials for subsequent sequencing and virus detection process through RT-PCR.

### High-throughput sequencing

The total RNA of each species was extracted from one plant using the EASY spin Plus Complex Plant RNA Kit (Aidlab, Beijing, China). The RNA purity, concentration, and integrity were evaluated using a Nanodrop (Thermo Fisher Scientific, Cleveland, OH, USA), Qubit 3.0 (Invitrogen, Waltham, MA, USA), and Agilent2100 (plant RNA Nano Chip, Agilent, Santa Clara, CA, USA), respectively. The ribosome RNA was depleted by the RiboZero Magnetic Kit (Epicenter, Madison, WI, USA), and a library was then built using a TruSeq RNA Sample Prep Kit (Illumina, San Diego, CA, USA). Furthermore, RNA-seq was conducted by Beijing Genomics Institution (BGI) using the BGI500 platform set 100 bp for the length of paired-end (PE) reads, by Mega Genomics (MG, Beijing, China) using an Illumina HiSeq X-Ten platform (PE 150 bp), or by Berry Genomics Corporation (BGC, Beijing, China) with an Illumina NovaSeq 6000 platform (PE 150 bp).

### BLAST analysis

The HTS data generated were processed through assembly using the CLC Genomics Workbench 11 (Supplementary Table S2). Prior to assembly, several steps were taken to enhance data quality, including the removal of adaptors and elimination of low-quality [proportion of low-quality base (quality score < 5) in single read > 20 per cent] and N-rich reads (number of N base in single read > 3), using the fastp tool in OpenGene package (https://opengene.org/). Where possible, draft genomes of related plant species were employed as references to filter out host-related reads from the dataset, thereby minimizing host genetic interference. The assembled contigs were first annotated using the prebuilt non-redundant protein sequences (nr) database dedicated to viruses (taxid:10239) and local BLASTx (e-value cutoff: 1e-4) of the fast-speed DIAMOND (Buchfink, Reuter, and Drost et al. 2021). The annotated putative viral contigs were used as reference sequences to build a database for subsequent iBLASTx (e-value cutoff: 1e-4) analysis of the unannotated contigs using the BLAST (version = 2.12.0+) -based iBLASTx tool. Then, the remaining unknown contigs as query and the annotated putative viral contigs from both the BLASTx and iBLASTx analyses as references were used for UTR-iBLASTn (e-value cutoff: 1e-2) analysis. For more online data in NCBI, the verified sequences of the putative new viruses through cloning and Sanger sequencing (Supplementary Table S3) were subjected to online BLASTn and BLASTx searches (e-value cutoff: 1e-4) against the TSA (higher plants, taxid:3193) and nr/nt databases, and new viral sequences as outcomes were used as new queries until there were no additional results. The SRA datasets of certain viruses identified from the online BLAST were downloaded and processed, as was done for the sequencing data of sampled plants. The NCBI-BLAST searches were accessed in years of 2019 and 2020. The local BLASTx/iBLAST and online BLASTx/BLASTn results with the threshold e-values were further analysed, and false positive results removed through sequence homology analysis. For a specific virus and its potential segments, the analysis includes searching for conserved nucleotides at the genomic termini (> eight consecutive identical nucleotides) through sequence alignment and subsequently calculating nucleotide sequence identity (> 50 per cent, no gaps) between UTRs, and finding conserved protein sequences (e-value < 1e-4) through BLASTp if 5′- and 3′-terminal sequences of a genomic RNA are not conserved enough. Such analysis was conducted only once at the final step. All the obtained putative viral contigs and TSA sequences were loaded into a final reciprocal BLASTx test and their UTRs extracted for a reciprocal BLASTn test using local programs of the BLAST-2.12.0+, with a default e-value. With all the analyses above, the ORSs were determined. Note that data of each sample were separately analysed but with the same procedures.

### UTR networks of viral contigs and TSA sequences

Based on the results of local reciprocal BLASTx and BLASTn, the putative genomic RNA sequences of each virus were separately networked by the Cytoscape 3.8.0 (Smoot et al. 2011) with Attribute Circle Layout that arranged them by the RNA names and with removing nonsignificant edges (BLASTn e-value > 1.6e-3) between the nodes that represent the RNAs (Fig. 3).

### Recovery of viral sequences

Specific primer pairs were designed at appropriate positions of the putative viral contigs using the CLC Genomics Workbench 11 and the Primer Premier 5.0 (Premier Biosoft, Palo Alto, CA, USA) to amplify overlapping fragments (Supplementary Table S4). Then, a one-step reverse transcription-PCR (RT-PCR) assay was conducted with a PrimeScript kit (Takara, Tokyo, Japan), and viral genomic terminal sequences were determined using commercial 5′ and 3′ RACE (rapid amplification of cDNA ends) kits (Invitrogen, Waltham, MA, USA). The PCR products were gel-purified using the Gel Extraction Kit (OMEGA Bio-Tec Inc., Doraville, GA, USA) and cloned on pEASY-T1 vectors (TransGen, Beijing, China) using competent cells. Five clones of each amplicon were fully sequenced with primer in both directions (Tsingke, Chengdu, China), and the output sequences were *de novo* assembled in the SeqMan program (DNAStar, Madison, WI, USA).

### Field investigation

Virus-specific detection primers were designed in conserved coding regions of the putative viral genomes using the same programs mentioned above. To avoid high viral heterogeneity among different samples, we collected plants within a limited area in an orchard, garden, or field, less than 0.01 km^2^ in size. The replication of viral ORSs, D-RNA, and subvirus relied on a compatible RdRP: therefore, we first detected the RdRP-associated RNAs in samples by RT-PCR, and the positive samples were further tested for the presence of ORSs, D-RNA, and satellite (Table 1).

### Viral small RNA profiles

In sRNA-seq, total sRNA was extracted from leaf tissues with the EASYspin Plant microRNA Extract kit (Aidlab, Beijing, China). The sRNA library was constructed using a TruSeq Small RNA Sample Prep Kit (Illumina, San Diego, CA, USA). Sequencing was performed using Illumina Hiseq2500 platform (MG, Beijing, China), NextSeq CN500 (BGC, Beijing, China), and BGI500 (BGI, Beijing, China) platforms. The resulting sRNAs were processed to remove adaptors, poly(A), low quality (limit: 0.05) and N-containing reads, and the remaining reads directly mapped on the HTS contig sequences as references using the CLC Genomics Workbench 11. Through the statistical analysis, putative viral contigs with sRNAs distribution characteristics in the size and 5′-nt being similar to the known viral sequences were collected. The data of the distributions of vsiRNAs from all of the multipartite-like viral contigs were normalized and visualized with the heatmap.2 function of gplots package 3.1.0 in R. The normality (Gaussian distribution) of the size distribution and 5′-nt preference of visRNAs were tested by the D’Agostino & Pearson method in GraphPad Prism 8.0 (Graph Pad Software, San Diego, CA, USA). The correlation coefficient was evaluated by both the Pearson (r_p_) and Spearman (r_s_) methods in GraphPad Prism 8.0 (Fig. 4).

### Sequence analysis

The ORFs of viral nucleotide sequences and conserved domains in protein sequences inferred from ORFs were found using the NCBI ORF finder (https://www.ncbi.nlm.nih.gov/orffinder) and CDD (https://www.ncbi.nlm.nih.gov/Structure/cdd/wrpsb.cgi) servers, respectively. The ORSs were reanalysed using the DIAMOND-BLASTx with the local nr database. Within-group relationships of the ORSs from each JMV, JVV, CNV, and BNV/VGV group were reestablished by local BLASTx-2.12.0+ with 1e-4 as the threshold e-value to search for their homologs. All these steps were accomplished for viral sequences to identify known genes/segments, gene duplication or duplicate segment homologs, and the HGTs from non-viral organisms (Table 2, Supplementary Fig. S1, S3, S5, S6, Table S3). The viral amino acid sequences selected were compared, and identities were calculated using the CLC Genomics Workbench 11.

### Phylogenetic analysis

For the tree of the phylum *Kitrinoviricota*, representative viral RdRP sequences of the order *Amarillovirales*, as shown by Paraskevopoulou et al. (2021), were retrieved from NCBI using the Batch Entrez server (https://www.ncbi.nlm.nih.gov/sites/batchentrez). Viral genomes of the order *Martellivirales* (taxonomy ID: 2,732,544) were obtained from the NCBI databases, and the RdRP sequences were identified using the Batch CD-Search server (https://www.ncbi.nlm.nih.gov/Structure/bwrpsb/bwrpsb.cgi) and extracted by the Strawberry Perl (5.32.1.1) using a script (Supplementary Data S2). All of the RdRPs were aligned by MAFFT v7.471 (Katoh and Standley 2013), using the E-INS-i algorithm, and then trimmed by trimAl 1.2rev57 (Capella-Gutiérrez, Silla-Martínez, and Gabaldón et al. 2009), with the automated1 algorithm to remove poorly aligned regions (Supplementary Data S3). Then, phylogenetic relationships of the remaining alignments were computed by the FastTree 2.1.11 (Price et al. 2010) with the LG + CAT model (Fig. 5). The other viral protein sequences were preprocessed by the same methods, but the tree files were generated using the IQ-TREE 1.6.12 (Nguyen et al. 2015), with the best-suited model automatically selected by the component tool according to the Bayesian Information Criterion and 1,000 bootstrap replicates (Fig. 6, 7 and Supplementary Fig. S2, S4). All the tree files were finally modified by the FigTree v1.4.4 (http://tree.bio.ed.ac.uk/software/figtree/).

### Evolution of the UTRs

For the family *Kitaviridae* and the RSVGV, 3′ UTRs of each virus were separately aligned by the MAFFT (E-INS-i) and trimmed by the trimAl (automated1). This was done to obtain conserved signals specific to each virus and to avoid false phylogenetic connections between any two viruses. Note that putative coding sequences behind the fourth ORF in RNA3 of PTBV were also included in the 3′ UTR for phylogenetic analyses. All the trimmed alignments were merged for realignment and analysed through the workflows of the same MAFFT algorithm, IQ-TREE with the recommended model, and 1,000 replicates of bootstrap; FigTree was used to draw and modify the phylograms Local BLASTn-2.12.0+ with the parameter not filtering low-complexity regions was executed for all the viral UTRs that are used as both queries and targets. The yFiles Radial Layout in the Cytoscape was used to display networks of the UTRs as nodes with the e-values as edges (1e-4 as the cutoff for visible) (Fig. 8).

## Supplementary Material

veae004_Supp

## Data Availability

All data obtained from this study are available in the main text, the supporting information, and the NCBI databases with GenBank accession numbers of OL344024–OL344048, BK061344–BK061350, OP807958–OP807963, OM687521–OM687525, MN915109–MN915111, and OR532447–OR532448, and with BioProject and SRA accession numbers recorded in Supplementary Table S2.
