# Supplementary material for: Conserved untranslated regions of multipartite viruses: Natural markers of novel viral genomic components and tags of viral evolution
Source: Virus Evol. 2024 Jan 12;10(1):veae004. doi: 10.1093/ve/veae004 (PMC10868557; doi:10.1093/ve/veae004)
Supplement: veae004_Supp [file veae004_supp.zip › suppl_data/Supplementary Table S1 to S4.docx]

Table S1 List of representative multipartite/segmented viruses, conserved UTRs of each tested by reciprocal BLASTn, multiple sequence alignment, and pairwise comparison.

| **Genome** | **Family** | **Genus** | **Virus** | **Conserved UTR** | **UTR/IR length (nt)** | **BLASTn E-value** | **Partial alignment (nt)** | **Pairwise identity in alignment** |
| --- | --- | --- | --- | --- | --- | --- | --- | --- |
| ssDNA | *Geminiviridae* | *Begomovirus* | Bean golden yellow mosaic virus (DQ119824, DQ119825) | IR | 361, 923 | 3.83e-72, 1.47e-72 | 366 | 80.6% |
|  | *Nanoviridae* | *Babuvirus* | Abaca bunchy top virus (NC_010314-NC_010319) | IR | 238-1,057 | 5.5e-11–3.32e-120 | 568 | 20.4-83.9% |
|  |  | *Nanovirus* | Subterranean clover stunt virus (NC_003812-NC_003819) | IR | 144-662 | 6e-2–5.67e-160 | 231 | 21.9-94.3% |
| +ssRNA | *Bromoviridae* | *Alfamovirus* | Alfalfa mosaic virus (L00163, X01572, K02703) | 3′ | 163-179 | 3.06e-47–2.76e-66 | 168 | 79.1-89.2% |
|  |  | *Anulavirus* | Pelargonium zonate spot virus (NC_003649-NC_003651) | 3′ | 290-416 | 9.84e-94–5.58e-114 | 299 | 81.6-88.3% |
|  |  | *Bromovirus* | Brome mosaic virus (GU584129-GU584131) | 3′ | 274-297 | 2.76e-91–5.64e-100 | 276 | 79-84.1% |
|  |  | *Cucumovirus* | Cucumber mosaic virus (NC_001440, NC_002034, NC_002035) | 3′ | 281-303 | 5.21e-107–1.06e-115 | 283 | 89-91.5% |
|  |  | *Ilarvirus* | Citrus variegation virus (NC_009536-NC_009538) | 3′ | 130-327 | 3.51e-29–6.75e-89 | 131 | 80.1-95.3% |
|  |  | *Oleavirus* | Olive latent virus 2 (NC_003671, NC_003673, NC_003674) | 3′ | 258-445 | 1.49e-78–3.61e-96 | 257 | 82.1-89.2% |
|  | *Closteroviridae* | *Crinivirus* | Potato yellow vein virus (NC_006061-NC_006063) | 3′ | 233-288 | 6.32e-10–2.79e-51 | 238 | 58.4-76% |
|  | *Kitaviridae* | *Blunervirus* | Tea plant necrotic ring blotch virus (NC_040401-NC_040404) | 3′ | 155-956 | 2.1e-7–4.18e-32 | 151 | 45.7-71% |
|  |  | *Cilevirus* | Citrus leprosis virus C (NC_008169, NC_008170) | 3′ | 244, 249 | 1.9e-89, 1.95e-89 | 250 | 86.8% |
|  |  | *Higrevirus* | Hibiscus green spot virus 2 (NC_016141-NC_016143) | 3′ | *198-223 | 9.55e-12–3.82e-16 | 181 | 59.1-64.7% |
|  | *Mayoviridae* | *Pteridovirus* | Japanese holly fern mottle virus (NC_013133, NC_013134) | 5′&3′ | 45&68, 75&70 | 3.3e-23, 2.57e-23 | 117 | 77.8% |
|  | *Virgaviridae* | *Furovirus* | Soil-borne wheat mosaic virus (NC_002041, NC_002042) | 3′ | 404, 463 | 2.62e-43, 2.28e-43 | 395 | 65.6% |
|  |  | *Goravirus* | Gentian ovary ringspot virus (NC_024501, NC_024502) | 3′ | 278, 307 | 6.76e-91, 6.09e-91 | 278 | 83.5% |
|  |  | *Hordeivirus* | Barley stripe mosaic virus (NC_003469, NC_003478, NC_003481) | 3′ | 256-258 | 3.34e-113–7.8e-135 | 258 | 98.8-99.6% |
|  |  | *Pecluvirus* | Peanut clump virus (NC_003668, NC_003672) | 3′ | 276, 298 | 4.04e-125, 3.73e-125 | 277 | 94.6% |
|  |  | *Pomovirus* | Potato mop-top virus (NC_003723-NC_003725) | 3′ | 343-490 | 4.89e-130–2.89e-139 | 343 | 87.7-90.1% |
|  |  | *Tobravirus* | Tobacco rattle virus (NC_003805, NC_003811) | 5′&3′ | 202&255, 556&390 | 1.95e-114–7.29e-115 | 457 | 85.6% |
|  | *Alphatetraviridae* | *Omegatetravirus* | Helicoverpa armigera stunt virus (NC_001981, NC_001982) | 3′ | 161, 169 | 1.45e-23, 1.38e-23 | 163 | 69.9% |
|  | *Potyviridae* | *Bymovirus* | Barley yellow mosaic virus (MF919136, MF919137) | 5′ | 154, 168 | 1.23e-49, 1.12e-49 | 154 | 81.8% |
|  | *Secoviridae* | *Comovirus* | Cowpea mosaic virus (NC_003549, NC_003549) | 5′&3′ | 160&180, 206&82 | 1.96e-15, 2.33e-15 | 298 | 61.7% |
|  |  | *Fabavirus* | Broad bean wilt virus 2 (KX686589, KX686590) | 5′&3′ | 227&179, 235&110 | 7.9e-36, 6.68e-36 | 309 | 73.8% |
|  |  | *Nepovirus* | Tomato ringspot virus (NC_003839, NC_003840) | 5′&3′ | 75&1,543, 75&1,547 | 0 | 1,622 | 99.2% |
|  |  | *Cheravirus* | Cherry rasp leaf virus (AJ621357, AJ621357) | 5′&3′ | 139&142, 292&140 | 4.71e-64, 3.02e-64 | 281 | 80.4% |
|  |  | *Sadwavirus* | Satsuma dwarf virus (AB009958, AB009959) | 5′&3′ | 300&248, 301&318 | 2.81e-109, 2.49e-109 | 554 | 83.9% |
|  |  | *Torradovirus* | Tomato torrado virus (KX132808, KX132809) | 5′&3′ | 107&1,069, 181&1,105 | 0 | 1,181 | 94.7% |
| -ssRNA | *Rhabdoviridae* | *Varicosavirus* | Lettuce big-vein associated virus (NC_011558, NC_011568) | 3′ | 336, 445 | 5.31e-64, 3.99e-64 | 338 | 66.3% |
| dsRNA | *Chrysoviridae* | *Alphachrysovirus* | Penicillium chrysogenum virus (AF296439-AF296442) | 5′ | 144-162 | 4.34e-14–5.14e-51 | 164 | 50-84.5% |
|  |  | *Betachrysovirus* | Botryosphaeria dothidea chrysovirus 1 (KF688736-KF688739) | 5′ | 230-293 | 3.17e-15–9.07e-54 | 272 | 47.2-70.6% |
|  | *Quadriviridae* | *Quadrivirus* | Rosellinia necatrix quadrivirus 1 (NC_016757-NC_016760) | 3′ | 94-425 | 1.32e-14–6.91e-39 | 125 | 51.6-84.8% |
|  | *Megabirnaviridae* | *Megabirnavirus* | Rosellinia necatrix megabirnavirus 1 (AB512282, AB512283) | 5′ | 1,636, 1,656 | 0 | 1,597 | 73.8% |
|  | *Cystoviridae* | *Cystovirus* | Pseudomonas phage phi6 (NC_003714-NC_003716) | 3′ | 114-666 | 2.06e-22–1.59e-33 | 118 | 71.2-83.4% |
|  | *Partitiviridae* | *Alphapartitivirus* | Beet cryptic virus 1 (NC_011556, NC_011557) | 5′ | 93, 122 | 1.19e-20–8.82e-21 | 100 | 75% |
|  |  | *Cryspovirus* | Cryptosporidium parvum virus 1 (NC_038843, NC_038844) | 3′ | 131, 188 | 1.47e-17–9.99e-18 | 141 | 63.1% |
|  | *Orthornavirae* | *Botybirnavirus* | Botrytis porri botybirnavirus 1 (NC_017990, NC_017991) | 5′ | 403, 404 | 0 | 404 | 94.8% |

Note: * Partial putative coding sequence was included in UTR of RNA3 (198 nt) for analysis.

Table S2 Information of sequencing data from sampled plants or the NCBI databases, and the results of data processing.

| **Source** | **Potential host** | **Sample** | **BioProject** | **SRA** | **RNA-seq platform** | **Clean reads** | **Non-host reads** | **No. of contigs** | **Contig length** | **Multipartite-like viral contigs** | **iBLASTx** | **iBLASTn (ORS)** ^b^ | **Virus type** | **Virus taxonomy** | **sRNA-seq platform** | **sRNA-seq clean reads** | **SRA** |
| --- | --- | --- | --- | --- | --- | --- | --- | --- | --- | --- | --- | --- | --- | --- | --- | --- | --- |
| Field sampling | Loquat | B444 | PRJNA1055000 | SRR27295524 | Illumina NovaSeq 6000 | 70,959,296 (9.91 Gb) | 2,441,384 (3.44%) | 48,785 | 200–8,126 | 5 | 2 | 3 | JMV ^c^ | *Amarillovirales* | NextSeq CN500 | 15,099,953 (14.4 M) | SRR27295523 |
| Field sampling | Ailanthus | CH-GZ | PRJNA1054777 | SRR27292984 | Illumina NovaSeq 6000 | 102,576,226 (14.33 Gb) | - | 122,063 | 200–23,578 | 11 | 6 | 5 | BNV/JMV | *Martellivirales*/*Amarillovirales* | NextSeq CN500 | 12,450,396 (11.87 M) | SRR27292983 |
| Field sampling | Apple | A68 | PRJNA1054979 | SRR27295021 | Illumina HiSeq X-ten | 73,291,484 (10.24 Gb) | 2,899,424 (3.96%) | 58,372 | 200–2,4032 | 5 | 4 | 1 | BNV | *Martellivirales* | Illumina Hiseq2500 | 18,008,805 (17.17 M) | SRR27295020 |
| Field sampling | Camellia | SC-L16 | PRJNA1054964 | SRR27295914 | Illumina HiSeq X-ten | 77,642,166 (10.84 Gb) | 3,352,543 (4.32%) | 15,876 | 200–8,750 | 5 | 4 | 1 | BNV | *Martellivirales* | Illumina Hiseq2500 | 36,431,857 (34.74 M) | SRR27295913 |
| Field sampling | Paper mulberry | PX | PRJNA1054806 | SRR27295107 | Illumina HiSeq X-ten | 59,993,834 (8.38 Gb) | 11,874,493 (19.8%) | 105,492 | 200–8,863 | 4 | 4 | 0 | CNV | *Martellivirales* | - | - | - |
| Field sampling | Jasmine | JY | PRJNA1054816 | SRR27294325 | BGI500 | 101,383,762 (9.44 Gb) | - | 44,423 | 200–13,257 | 5 | 4 | 1 | CNV/SLV | *Martellivirales/*Satellite virus | BGI500 | 39,008,596 (37.2 M) | SRR27295024 |
| Field sampling | Citrus | B2-20R | PRJNA1054798 | SRR27293115 | Illumina NovaSeq 6000 | 64,029,758 (8.94 Gb) | 1,947,728 (3.42%) | 3,660 | 200–19,173 | 6 | 3 | 3 | JVV | *Martellivirales* |  | - | - |
| TSA ^a^ | *Fagus crenata* | - | PRJNA523682 | - | - | - | - | - | - | 4 | 2 | 2 | JMV | *Amarillovirales* | - | - | - |
| SRA | Grape, *Plasmopara viticola* | - | PRJNA613358 | SRR11364892 | - | - | - | - | - | 4 | 2 | 2 | JMV | *Amarillovirales* | - | - | - |
| SRA | *Phalaenopsis equestris* | - | PRJNA288388 | SRR2080204 | - | - | - | - | - | 4 | 2 | 2 | JMV | *Amarillovirales* | - | - | - |
| TSA | *Paulownia tomentosa* | - | PRJNA285426 | - | - | - | - | - | - | 4 | 3 | 1 | BNV | *Martellivirales* | - | - | - |
| SRA | *Camellia sinensis* | - | PRJNA352285 | SRR6459579 | - | - | - | - | - | 1 | 0 | 1 | BNV | *Martellivirales* | - | - | - |
| TSA | *Rhazya stricta* | - | PRJNA215242 | - | - | - | - | - | - | 5 | 1 | 4 | VGV | *Martellivirales* | - | - | - |
| TSA | *Carya illinoinensis* | - | PRJNA411951 | - | - | - | - | - | - | 15 | 9 | 6 | JVV | *Martellivirales* | - | - | - |
| SRA | Grape, *Plasmopara viticola* | - | PRJNA558612 | SRR9995128 | - | - | - | - | - | 2 | 0 | 2 | JVV | *Martellivirales* | - | - | - |
| TSA | *Picea glauca* | - | PRJNA210511 | - | - | - | - | - | - | 5 | 3 | 2 | JVV | *Martellivirales* | - | - | - |
| TSA | *Pinus flexilis* | - | PRJNA315892 | - | - | - | - | - | - | 5 | 3 | 2 | JVV | *Martellivirales* | - | - | - |
| GenBank | *Pistacia lentiscus* | - | - | - | - | - | - | - | - | 3 | 3 | 0 | JVV | *Martellivirales* | - | - | - |
| TSA | *Sarcandra glabra* | - | PRJNA671629 | - | - | - | - | - | - | 4 | 3 | 1 | JVV | *Martellivirales* | - | - | - |

Note: -, not available or important; ^a^ TSA (Transcriptome Shotgun Assembly), SRA (Sequence Read Archive), and GenBank databases of the NCBI; ^b^ ORS, orphan sequence; ^c^ JMV (jingmen-related virus), BNV (bluner-related virus), CNV (crini-related virus), SLV (satellite-related virus), JVV (jivi-related virus), and VGV (virga-related virus).

Table S3 Information of the viral sequences, and the results of sequence analyses.

| **Virus type** | **Virus/satellite** | RNA | **Size (nt)** | **GenBank or TSA** | **BioProject** | **SRA** | **CDD analysis (hit accession, e-value)** | **BLASTx with the nr (e-value < e-4)** | **local BLASTp (e-value < e-4)** | |
| --- | --- | --- | --- | --- | --- | --- | --- | --- | --- | --- |
| Jingmenvirus | AJMV1 | RNA1* | 3,315 | OP807958/*Data S1* | PRJNA1054777 | SRR27292984 | Capping_2-OMTase_viral (cl41719, 7.38e-29); Flavi_NS5 (cl03045, 9.15e-27) | Jingmenvirus | Plant/fungal Jingmenvirus | |
|  |  | RNA2a* | 2,862 | OP807960/*Data S1* | PRJNA1054777 | SRR27292984 | - | - | Plant/fungal Jingmenvirus | |
|  |  | RNA2b* | 2,843 | OP807961/*Data S1* | PRJNA1054777 | SRR27292984 | - | - | Plant/fungal Jingmenvirus | |
|  |  | RNA3* | 2,929 | OP807959/*Data S1* | PRJNA1054777 | SRR27292984 | DEAD-like_helicase_N and _C (cl28899, 1.02e-15, and cl38915, 5.65e-11, respectively) | Jingmenvirus | Plant/fungal Jingmenvirus | |
|  |  | RNA4* | 2,341 | OP807962/*Data S1* | PRJNA1054777 | SRR27292984 | - | - | - | |
|  |  | RNA5* | 1,717 | OP807963/*Data S1* | PRJNA1054777 | SRR27292984 | - | - | - | |
|  | LJMV1 | RNA1 | 3,323 | OL344024 | PRJNA1055000 | SRR27295524 | Capping_2-OMTase_viral (cl41719, 3.48e-27); Flavi_NS5 (cl03045, 1.13e-26) | Jingmenvirus | Plant/fungal Jingmenvirus | |
|  |  | RNA2 | 3,218 | OL344025 | PRJNA1055000 | SRR27295524 | pRha (cl34654, 5.42e-3) ^a^ | - | Plant/fungal Jingmenvirus | |
|  |  | RNA3 | 3,193 | OL344026 | PRJNA1055000 | SRR27295524 | DEAD-like_helicase_N and _C (cl28899, 2.12e-12, and cl38915, 1.55e-09, respectively) | Jingmenvirus | Plant/fungal Jingmenvirus | |
|  |  | RNA4 | 2,825 | OL344027 | PRJNA1055000 | SRR27295524 | - | - | Plant/fungal Jingmenvirus | |
|  |  | RNA5 | 2,216 | OL344028 | PRJNA1055000 | SRR27295524 | - | - | - | |
|  | FCJMV | RNA1 (TSA) | 1,234 | GHGX01021309.1 | PRJNA523682 | - | RT_like (cl02808, 2.49e-09) | Jingmenvirus | Plant/fungal Jingmenvirus | |
|  |  | RNA2 (TSA) | 1,756 | GHGX01197396.1 | PRJNA523682 | - | - | - | Plant/fungal Jingmenvirus | |
|  |  | RNA3 (TSA) | 1,852 | GHGX01147146.1 | PRJNA523682 | - | DEAD-like_helicase_N and _C (cl28899, 3.64e-16, and cl38915, 5.94e-06, respectively) | Jingmenvirus | Plant/fungal Jingmenvirus | |
|  |  | RNA4 (TSA) | 2,799 | GHGX01127647.1 | PRJNA523682 | - | - | - | Plant/fungal Jingmenvirus | |
|  | PEJMV | RNA1 (TSA) | 2,829 | GDHJ01006664.1 | PRJNA288388 | - | Capping_2-OMTase_viral (cl41719, 6.21e-27); Flavi_NS5 (cl03045, 6.26e-27) | Jingmenvirus | Plant/fungal Jingmenvirus | |
|  |  | RNA2 (SRA) | 2,515 | BK061344 | PRJNA288388 | SRR2080204 | - | - | Plant/fungal Jingmenvirus |  |
|  |  | RNA3 (TSA) | 3,364 | GDHJ01005091.1 | PRJNA288388 | - | DEAD-like_helicase_N and _C (cl28899, 5.86e-23, and cl38915, 4.08e-12, respectively) | Jingmenvirus | Plant/fungal Jingmenvirus | |
|  |  | RNA4 (SRA) | 3,261 | BK061345 | PRJNA288388 | SRR2080204 | - | - | Plant/fungal Jingmenvirus | |
|  | PVaJMV1 | RNA1 (SRA/GenBank) | 3,243 | *Data S1*/MN551116 | PRJNA613358 | SRR11364892 | Capping_2-OMTase_viral (cl41719, 8.91e-35); Flavi_NS5 (cl03045, 1.72e-23) | Jingmenvirus | Plant/fungal Jingmenvirus | |
|  |  | RNA2 (SRA) | 3,006 | BK061346 | PRJNA613358 | SRR11364892 | - | - | Plant/fungal Jingmenvirus | |
|  |  | RNA3 (GenBank) | 3,002 | MN551114 | - | - | DEAD-like_helicase_N and _C (cl28899, 1.87e-15, and cl38915, 3.35e-15, respectively) | Jingmenvirus | Plant/fungal Jingmenvirus | |
|  |  | RNA4 (SRA) | 2,732 | BK061347 | PRJNA613358 | SRR11364892 | - | - | Plant/fungal Jingmenvirus | |
| Blunervirus | AiBV1 | RNA1* | 7,493 | OM687521 | PRJNA1054777 | SRR27292984 | Vmethyltransf (cl03298, 8.36e-11); Viral_helicase1 (cl26263, 5.73e-24); RdRP_2 (cl03049, 0) | Blunervirus | Blunervirus | |
|  |  | RNA2* | 6,107 | OM687522 | PRJNA1054777 | SRR27292984 | Vmethyltransf (cl03298, 1.08e-07); Viral_helicase1 (pfam01443, 1.42e-30) | Blunervirus | Blunervirus | |
|  |  | RNA3* | 3,941 | OM687523 | PRJNA1054777 | SRR27292984 | Viral_helicase1 (pfam01443, 2.16e-31); RdRP_2 (cl03049, 2.61e-70) | Blunervirus | Blunervirus | |
|  |  | RNA4* | 3,348 | OM687524 | PRJNA1054777 | SRR27292984 | SP24 (pfam16504, 3.67e-18); PHA02030 (cl25717, 1.89e-3) | Blunervirus | Blunervirus | |
|  |  | RNA5* | 2,712 | OM687525 | PRJNA1054777 | SRR27292984 | - | - | Blunervirus | |
|  | ApBV1 | RNA1* | 6,014 | OL344039 | PRJNA1054979 | SRR27295021 | Vmethyltransf (cl03298, 4.72e-06); Viral_helicase1 (pfam01443, 1.42e-29) | Blunervirus | Blunervirus | |
|  |  | RNA2* | 3,868 | OL344040 | PRJNA1054979 | SRR27295021 | Viral_helicase1 (pfam01443, 1.84e-34); RdRP_2 (cl03049, 8.92e-77) | Blunervirus | Blunervirus | |
|  |  | RNA3* | 3,433 | OL344041 | PRJNA1054979 | SRR27295021 | SP24 (pfam16504, 8.63e-19) | Blunervirus | Blunervirus | |
|  |  | RNA4* | 2,823 | OL344042 | PRJNA1054979 | SRR27295021 | - | - | Blunervirus | |
|  |  | RNA5* | 2,268 | OL344043 | PRJNA1054979 | SRR27295021 | Prominin (cl25943, 4.67e-3) | - | Blunervirus | |
|  | TPNRBV-Ca1 | RNA1* | 5,925 | OL344044 | PRJNA1054964 | SRR27295914 | Vmethyltransf (cl03298, 2.49e-20); Viral_helicase1 (cl26263, 1.89e-16) | Blunervirus | Blunervirus |  |
|  |  | RNA2* | 4,129 | OL344045 | PRJNA1054964 | SRR27295914 | Viral_helicase1 (cl26263, 1.08e-24); RdRP_2 (cl03049, 2.28e-82) | Blunervirus | Blunervirus |  |
|  |  | RNA3* | 2,732 | OL344046 | PRJNA1054964 | SRR27295914 | SP24 (pfam16504, 1.31e-25) | Blunervirus | Blunervirus |  |
|  |  | RNA4* | 2,179 | OL344047 | PRJNA1054964 | SRR27295914 | 3A (6.11e-20) | Blunervirus | Blunervirus |  |
|  |  | RNA5* | 2,284 | OL344048 | PRJNA1054964 | SRR27295914 | - | - | Blunervirus |  |
|  | TPNRBV | RNA5 (SRA) | 2,288 | BK061350 | PRJNA352285 | SRR6459579 | - | - | Blunervirus | |
|  | PTBV | RNA1 (TSA) | 6,147 | GEFV01158142.1 | PRJNA285426 | - | Vmethyltransf (cl03298, 1.31e-10); Viral_helicase1 (cl26263, 2.18e-27) | Blunervirus | Blunervirus | |
|  |  | RNA2 (TSA) | 3,678 | GEFV01018191.1 | PRJNA285426 | - | Viral_helicase1 (pfam01443, 8.00e-26); RdRP_2 (cl03049, 1.79e-76) | Blunervirus | Blunervirus | |
|  |  | RNA3 (TSA) | 3,399 | GEFV01018861.1 | PRJNA285426 | - | SP24 (pfam16504, 7.08e-17) | Blunervirus | Blunervirus | |
|  |  | RNA4 (TSA) | 3,003 | GEFV01018726.1 | PRJNA285426 | - | PHA03247 (cl33720, 2.10e-3) | - | Blunervirus | |
|  | RSVGV | RNA1 (TSA) | 9,987 | GAMW01005974.1 | PRJNA215242 | - | Vmethyltransf (cl03298, 9.04e-24); Viral_helicase1 (cl26263, 2.02e-33); RdRP_2 (pfam00978, 4.71e-116); TMV_coat (cl20208, 1.13e-3; 1.72e-05; 4.40e-10) | Hubei virga-like virus 17 | Hubei virga-like virus 17 | |
|  |  | RNA2 (TSA) | 1,424 | GAMW01019114.1 | PRJNA215242 | - | - | - | - | |
|  |  | RNA3 (TSA) | 1,418 | GAMW01002782.1 | PRJNA215242 | - | - | - | - | |
|  |  | RNA4 (TSA) | 799 | GAMW01000142.1 | PRJNA215242 | - | - | - | - | |
|  |  | RNA5 (TSA) | 786 | GAMW01024581.1 | PRJNA215242 | - | - | - | - | |
| Crinivirus | PMCV1 | RNA1a* | 9,005 | OL344035 | PRJNA1054806 | SRR27295107 | 2OG-FeII_Oxy (cl21496, 2.77e-09); Vmethyltransf (pfam01660, 5.69e-31); Viral_helicase1 (cl26263, 5.18e-23); RdRP_2 (pfam00978, 1.80e-124) | Crinivirus | Crinivirus |  |
|  |  | RNA1b* | 9,005 | OL344036 | PRJNA1054806 | SRR27295107 | 2OG-FeII_Oxy (cl21496, 4.48e-09); Vmethyltransf; Viral_helicase1; RdRP_2 | Crinivirus | Crinivirus |  |
|  |  | RNA2* | 6,853 | OL344037 | PRJNA1054806 | SRR27295107 | HSP70_NBD (cd10170 , 3.66e-54); Viral_Hsp90 (pfam03225, 1.28e-148); Closter_coat (cl03354, 5.34e-39) | Crinivirus | Crinivirus |  |
|  |  | D-RNA* | 2,394 | OL344038 | PRJNA1054806 | SRR27295107 | HSP70_NBD; Viral_Hsp90 | Crinivirus | Crinivirus |  |
|  | JVA | RNA1a* | 8,237 | MN915109 | PRJNA1054816 | SRR27294325 | Vmethyltransf (pfam00978, 4.49e-24); Viral_helicase1 (cl26263, 2.17e-23); RdRP_2 (pfam00978, 3.78e-129) | Crinivirus | Crinivirus | |
|  |  | RNA1b* | 8,228 | MN915111 | PRJNA1054816 | SRR27294325 | Vmethyltransf; Viral_helicase1; RdRP_2 | Crinivirus | Crinivirus | |
|  |  | RNA2* | 8,003 | MN915110 | PRJNA1054816 | SRR27294325 | HSP70_NBD (cd10170 , 1.94e-50); Viral_Hsp90 (pfam03225, 7.67e-147); Closter_coat (cl03354, 2.06e-33) | Crinivirus | Crinivirus | |
|  |  | RNA3* | 1,191 | OR532447 | PRJNA1054816 | SRR27294325 | - | - | - | |
| Satellite virus | JVAaSV | RNA* | 1,211 | OR532448 | PRJNA1054816 | SRR27294325 | Potex_coat (cl05609, 9.66e-12) | Cassava satellite virus | Cassava satellite virus | |
| Jivivirus | CJVV1 | RNA1* | 4,061 | OL344029 | PRJNA1054798 | SRR27293115 | Vmethyltransf (cl03298, 8.29e-13); Viral_helicase1 (1.54e-33, pfam01443) | Jivivirus | Plant/fungal Jivivirus | |
|  |  | RNA2* | 3,236 | OL344030 | PRJNA1054798 | SRR27293115 | RdRP_2 (cl03049, 1.13e-75) | Jivivirus | Plant/fungal Jivivirus | |
|  |  | RNA3* | 2,185 | OL344031 | PRJNA1054798 | SRR27293115 | DEAD-like_helicase_N (cl28899, 2.02e-05) | Jivivirus | Plant/fungal Jivivirus | |
|  |  | RNA4* | 1,834 | OL344032 | PRJNA1054798 | SRR27293115 | - | - | Plant/fungal Jivivirus | |
|  |  | RNA5a* | 1,659 | OL344033 | PRJNA1054798 | SRR27293115 | ZnF_U1 (smart00451, 5.87e-05); zf-3CxxC (pfam13695, 3.97e-11) | Fungi (CEJ81816) | Plant/fungal Jivivirus | |
|  |  | RNA5b* | 1,656 | OL344034 | PRJNA1054798 | SRR27293115 | - | - | Plant/fungal Jivivirus | |
|  | CIJVV | RNA1a (TSA) | 2,434 | GGRT01036550.1 | PRJNA411951 | - | Viral_helicase1 (pfam01443, 7.97e-35) | Jivivirus | Plant/fungal Jivivirus | |
|  |  | RNA1b (TSA) | 2,542 | GGRT01047511.1 | PRJNA411951 | - | Viral_helicase1 (pfam01443, 6.57e-32) | Jivivirus | Plant/fungal Jivivirus | |
|  |  | RNA1c (TSA) | 2,689 | GGRT01059913.1 | PRJNA411951 | - | Viral_helicase1 (pfam01443, 1.25e-36) | Jivivirus | Plant/fungal Jivivirus | |
|  |  | RNA2a (TSA) | 3,176 | GGRT01000958.1 | PRJNA411951 | - | RdRP_2 (cl03049, 4.34e-62) | Jivivirus | Plant/fungal Jivivirus | |
|  |  | RNA2b (TSA) | 3,924 | GGRT01040104.1 | PRJNA411951 | - | RdRP_2 (cl03049, 1.06e-65) | Jivivirus | Plant/fungal Jivivirus | |
|  |  | RNA2c (TSA) | 2,246 | GGRT01068823.1 | PRJNA411951 | - | RdRP_2 (cl03049, 1.11e-62) | Jivivirus | Plant/fungal Jivivirus | |
|  |  | RNA3a (TSA) | 2,286 | GGRT01039285.1 | PRJNA411951 | - | - | Jivivirus | Plant/fungal Jivivirus | |
|  |  | RNA3b (TSA) | 2,184 | GGRT01046360.1 | PRJNA411951 | - | DEAD-like_helicase_N and _C (cl28899, 1.34e-4, and cl38915, 1.22e-4, respectively) | Jivivirus | Plant/fungal Jivivirus | |
|  |  | RNA3c (TSA) | 2,045 | GGRT01051128.1 | PRJNA411951 | - | DEAD-like_helicase_N (cl28899, 1.07e-4); DEXDc (smart00487, 5.70e-4) | Jivivirus | Plant/fungal Jivivirus | |
|  |  | RNA4a (TSA) | 1,832 | GGRT01011295.1 | PRJNA411951 | - | - | - | Plant/fungal Jivivirus | |
|  |  | RNA4b (TSA) | 1,978 | GGRT01039520.1 | PRJNA411951 | - | - | - | Plant/fungal Jivivirus | |
|  |  | RNA4c (TSA) | 1,793 | GGRT01044770.1 | PRJNA411951 | - | - | - | Plant/fungal Jivivirus | |
|  |  | RNA5a (TSA) | 2,217 | GGRT01023318.1 | PRJNA411951 | - | - | - | Plant/fungal Jivivirus | |
|  |  | RNA5b (TSA) | 1,543 | GGRT01045223.1 | PRJNA411951 | - | - | - | Plant/fungal Jivivirus | |
|  |  | RNA5c (TSA) | 1,287 | GGRT01048970.1 | PRJNA411951 | - | - | - | Plant/fungal Jivivirus | |
|  | GaJV1 | RNA4 (SRA) | 1,606 | BK061348 | PRJNA558612 | SRR9995128 | - | - | Plant/fungal Jivivirus | |
|  | GaJV2 | RNA4 (SRA) | 1,347 | BK061349 | PRJNA558612 | SRR9995128 | - | - | Plant/fungal Jivivirus | |
|  | MVY | RNA1 (GenBank) | 3,355 | MT334610 | - | - | - | Jivivirus | Plant/fungal Jivivirus | |
|  |  | RNA2 (GenBank) | 2,914 | MT334609 | - | - | RdRP_2 (cl03049, 8.14e-53) | Jivivirus | Plant/fungal Jivivirus | |
|  |  | RNA3 (GenBank) | 1,983 | MT334608 | - | - | - | Jivivirus | Plant/fungal Jivivirus | |
|  | PFJVV | RNA1 (TSA) | 3,869 | GHWE01373906.1 | PRJNA315892 | - | - | Jivivirus | Plant/fungal Jivivirus | |
|  |  | RNA2 (TSA) | 986 | GHWE01203419.1 | PRJNA315892 | - | - | Jivivirus | Plant/fungal Jivivirus | |
|  |  | RNA3 (TSA) | 2,472 | GHWE01351695.1 | PRJNA315892 | - | DEAD-like_helicase_N (cl28899, 2.17e-3) | Jivivirus | Plant/fungal Jivivirus | |
|  |  | RNA5a (TSA) | 1,933 | GHWE01320882.1 | PRJNA315892 | - | Nuc_deoxyri_tr2 (pfam15891, 2.31e-14) | Bacteria (WP_074674021) | Plant/fungal Jivivirus | |
|  |  | RNA5b (TSA) | 1,587 | GHWE01354688.1 | PRJNA315892 | - | - | - | Plant/fungal Jivivirus | |
|  | PGJVV | RNA1 (TSA) | 4,012 | GCHX01150549.1 | PRJNA210511 | - | - | Jivivirus | Plant/fungal Jivivirus | |
|  |  | RNA2 (TSA) | 3,101 | GCHX01086971.1 | PRJNA210511 | - | - | Jivivirus | Plant/fungal Jivivirus | |
|  |  | RNA3 (TSA) | 2,353 | GCHX01360593.1 | PRJNA210511 | - | DEAD-like_helicase_N (cl28899, 2.17e-3; 3.19e-3) | Jivivirus | Plant/fungal Jivivirus | |
|  |  | RNA4 (TSA) | 1,279 | GCHX01292299.1 | PRJNA210511 | - | - | - | Plant/fungal Jivivirus | |
|  |  | RNA5 (TSA) | 1,752 | GCHX01219314.1 | PRJNA210511 | - | Nuc_deoxyri_tr2 (pfam15891, 6.04e-14) | Bacteria (WP_091604020) | Plant/fungal Jivivirus | |
|  | SGJVV | RNA1 (TSA) | 3,962 | GJAT01123184.1 | PRJNA671629 | - | - | Jivivirus | Plant/fungal Jivivirus | |
|  |  | RNA2 (TSA) | 3,085 | GJAT01053493.1 | PRJNA671629 | - | - | Jivivirus | Plant/fungal Jivivirus | |
|  |  | RNA3 (TSA) | 2,008 | GJAT01025858.1 | PRJNA671629 | - | DEXDc (smart00487, 5.70e-4) | Jivivirus | Plant/fungal Jivivirus | |
|  |  | RNA4 (TSA) | 1,815 | GJAT01069010.1 | PRJNA671629 | - | - | - | Plant/fungal Jivivirus | |

Note: -, not available; * Genomic RNA with full-length sequence; ^a^ Red color indicates the possible horizontal gene transfer events.

Table S4 List of virus-primers used for verification of contig sequences and detection of the viruses in plant samples.

| **Primer type** | **Target** | **Name** | **Sequence (5’ to 3’)** | **Product size (bp)** |
| --- | --- | --- | --- | --- |
| genome recovery | ApBV1 RNA1 | AB1F1 | CACGTACCCCAACTATACCA | 1922 |
|  |  | AB1R1 | CAACCAAGCGAGCAATATCA |  |
|  |  | AB1F2 | TCTTCACACAACACGGCT | 1843 |
|  |  | AB1R2 | TGAACATAACGAAACTGGGG |  |
|  |  | AB1F3 | AACTGAAGAGCTGGACCAA | 1630 |
|  |  | AB1R3 | AGAATCCGGGTGGGTAAA |  |
|  |  | AB1GSP52 | TTCATCACCAAGCGTCTTTCCTC | 530 |
|  |  | AB1GSP32 | GATACGCCACCTAAATCCAAG | 590 |
|  | ApBV1 RNA2 | AB2F1 | GCTTTCGTTCGCTCTCAT | 1638 |
|  |  | AB2R1 | ATCACGACCACCTACCTT |  |
|  |  | AB2F2 | CTCGGTGGTGGAATAACT | 1890 |
|  |  | AB2R2 | ATCACGAGCCACACAAAC |  |
|  |  | AB2GSP52 | CGACGAACCTCAAAACCT | 453 |
|  |  | AB2GSP32 | GCGTGCGTTGTATGAGAGA | 351 |
|  | ApBV1 RNA3 | AB3F1 | GGAGAAGTAGGAGATGCAGAGA | 1614 |
|  |  | AB3R1 | CAAAGGACGAACGCAGGA |  |
|  |  | AB3F2 | CACGAAAACCCACCACCT | 1474 |
|  |  | AB3R2 | TTTCAACGGAGTCTGCCAC |  |
|  |  | AB3GSP52 | GAAGTATAAGCGCAAGCCGA | 339 |
|  |  | AB3GSP32 | AGATGCCGCCGTTGTGAGA | 394 |
|  | ApBV1 RNA4 | AB4F1 | TTACCGACCTTATTCGCA | 2332 |
|  |  | AB4R1 | TAAGCAACCGATACAGCA |  |
|  |  | AB4GSP52 | AGATGGTGAACTCGACGGTAA | 391 |
|  |  | AB4GSP32 | TTTTTCACCGACTATCCCCATT | 418 |
|  | ApBV1 RNA5 | AB5F1 | CAACACAACCCGAAAGCAAA | 1599 |
|  |  | AB5R1 | ACCAATCGGAACAAACCTCA |  |
|  |  | AB5GSP52 | CAGAAGCACCCGACGACAA | 435 |
|  |  | AB5GSP32 | GTCAAAGTGCTAATGGCGG | 480 |
|  | PMCV RNA1 | PC1F1 | CTCATCAACCCACCTTTCTACC | 1848 |
|  |  | PC1R1 | GAATCGTCCGTCAACTTCATCC |  |
|  |  | PC1F2 | GAGGGGTTTTGAAGAACAGAGT | 2065 |
|  |  | PC1R2 | TTGATAGTGGTCCTTCTGTTCG |  |
|  |  | PC1F3 | GCAAAGGCGGAGGTAAGTCTAT | 2011 |
|  |  | PC1R3 | GAATGAGCGTTCTTTACACCCT |  |
|  |  | PC1F4 | AAGGGTGTAAAGAACGCTCATT | 2026 |
|  |  | PC1R4 | GAATCTGACTGGGTCTGGAATG |  |
|  |  | PC1F5 | GGTGCTCAAAGAAGGACTGGAT | 1192 |
|  |  | PC1R5 | GATAACGCCGCATCTCTAAAAT |  |
|  |  | PC1GSP52 | AGTAACGGTTTGGGCATTCTATTGA | 443 |
|  |  | PC1GSP32 | GAAGCATACGCAAAAGAAGTTAGGA | 589 |
|  | PMCV RNA2 | PC2F1 | CCACGTGCTTGTCCATAACTAA | 1957 |
|  |  | PC2R1 | CCAAGTCATCCTCATCATCCAA |  |
|  |  | PC2F2 | GGACTTCAGTTAGGAGTGGCAA | 1910 |
|  |  | PC2R2 | CTTCTTCGTTCAAACAGTCTCC |  |
|  |  | PC2F3 | TCGGAGATGGACAACTAGAACC | 1984 |
|  |  | PC2R3 | GGATACCTCATCATTCTGGACG |  |
|  |  | PC2F4 | TACAACGTCCAGAATGATGAGG | 727 |
|  |  | PC2R4 | CACGCGTGTATGACTCTAAGGT |  |
|  |  | PC2GSP52 | TCTCAATAAAGATGGACGCGAAGGC | 242 |
|  |  | PC2GSP32 | GATGTCCTTGAGTTGAATAGGGGTT | 785 |
|  | CJVV1 RNA1 | CJ1GSP52 | TGAACTCGGGGAAAAGCATAGA | 403 |
|  |  | CJ1GSP32 | GTACAGATATAACACAGCGAAGTCG | 272 |
|  | CJVV1 RNA2 | CJ2GSP52 | CCAGAGGCTATCAAGACCAAAT | 387 |
|  |  | CJ2GSP32 | CCGGTTATGTCTTGCCTCTT | 376 |
|  | CJVV1 RNA3 | CJ3GSP52 | CAATGCTACCACAACCCTCTTA | 383 |
|  |  | CJ3GSP32 | AGACCGCAGCGTCATAGAGTTA | 514 |
|  | CJVV1 RNA4 | CJ4GSP52 | GTGGTTTCTCTCGTCGTCATCA | 447 |
|  |  | CJ4GSP32 | TTTGAATCTGCTTTGCTGGTCT | 189 |
|  | CJVV1 RNA5a | CJ5aGSP52 | ATGAAACTCTCGCGCTATATGC | 425 |
|  |  | CJ5aGSP32 | GTTTCACACGCTTCTGTTTCTACTG | 387 |
|  | CJVV1 RNA5b | CJ5bGSP52 | CAGATCCCCTTTTACTCGCAGA | 251 |
|  |  | CJ5bGSP32 | GTTGATTCGTAGCCGTTATTGC | 287 |
|  | TPNRBV RNA1 | TB1GSP52 | TCTATCCATCAGTTCTTTTGCG | 332 |
|  | TPNRBV RNA2 | TB2GSP52 | ATATGAATTCCTCGCCTAGCAA | 187 |
|  | TPNRBV RNA3 | TB3GSP52 | ACTGTTGAGACTACCAATCGCT | 252 |
|  |  | TB3GSP32 | GAAATAGCTGATACTGTCGGTG | 348 |
|  | TPNRBV RNA4 | TB4GSP52 | CCTCGTAAATTCTAGCGTCTGA | 218 |
|  |  | TB4F | TTGTCGGTGATGTGAAGTTAGC | 426 |
|  |  | TB4R | CTTAGAGTTGAAAACCCCACAG |  |
|  | TPNRBV RNA5 | TB5GSP52 | CTACCAGGAAATGTTACGGCTCTCA | 383 |
|  |  | TB5GSP32 | ATCTTGTCGATTGGAAAGGTGTAC | 361 |
| detection | ApBV1 RNA1 | AaBV-1-DF1 | CGTCCGAGTATTACCAGACTG | 702 |
|  |  | AaBV-1-DR1 | AATCACCGTCACCAGGAAC |  |
|  | ApBV1 RNA2 | AaBV-2-DF | TATGACACTTTGGCTTTGCTTG | 537 |
|  |  | AaBV-2-DR | ATAATCGTGACGTGCTTCCTTC |  |
|  | ApBV1 RNA3 | AaBV-3-DF | CGCTTGATGTGTTCTCAGG | 625 |
|  |  | AaBV-3-DR | AGTCCGTAGACCTTGCTCAG |  |
|  | ApBV1 RNA4 | AaBV-4-DF | CTCTGTTAGCCATTGTTCCATC | 568 |
|  |  | AaBV-4-DR | TTCAACAGTCCGTTAAAGTGGC |  |
|  | ApBV1 RNA5 | AaBV-5-DF | GTTTAGTGCTTTTACCAGTCGC | 754 |
|  |  | AaBV-5-DR | GTAACCGATACCGTCACCTAAC |  |
|  | TPNRBV RNA1 | Blu-RNA1-DF | TGTATCGGTCGAAGTGCC | 437 |
|  |  | Blu-RNA1-DR | TCAGTGTCGCAGAAACCC |  |
|  | TPNRBV RNA2 | Blu-RNA2-DF | AAAACCCGACGATTCTGA | 362 |
|  |  | Blu-RNA2-DR | CTTTCCGTTCAGCCACCT |  |
|  | TPNRBV RNA3 | Blu-RNA3-DF | GCGGAAAGGGACAAAGAT | 121 |
|  |  | Blu-RNA3-DR | AGCAAGAACGCTGACACG |  |
|  | TPNRBV RNA4 | Blu-RNA4-DF | AGACTGACTCTAAATGGGAAAG | 303 |
|  |  | Blu-RNA4-DR | CATCACCGACAACAGCGT |  |
|  | TPNRBV RNA5 | Blu-RNA5-DF | CGGTCTGGCTCCTATCTG | 273 |
|  |  | Blu-RNA5-DR | TGTAACCTGGTCCCCTTC |  |
|  | PMCV1 RNA1a | PC-1a-DF | TCAAACTGTCGTTCGTTCTG | 739 |
|  |  | PC-1a-DR | AATCACACCATTCACGGC |  |
|  | PMCV1 RNA1b | PC-1b-DF | TTATGGACCAACTCTCCGC | 520 |
|  |  | PC-1b-DR | GTTTCAACAGACTTCGCCAA |  |
|  | PMCV1 RNA2 | PC-2-DF | AGAACCCGATAAGTTGAAGGAC | 409 |
|  |  | PC-2-DR | ATGTTTCGCAGCCAGATG |  |
|  | PMCV1 RNA3 | PC-3-DF | GGGCGAATAACAGAGCAA | 208 |
|  |  | PC-3-DR | CCATCACTTTCTATCTTCCCAG |  |
|  | LJMV1 RNA1 | LJ-1-DF | ACCTCAGCCTCAACATCC | 545 |
|  |  | LJ-1-DR | TTTGCGCTCCTTCTTCCC |  |
|  | LJMV1 RNA2 | LJ-2-DF | AGCTGAAGTGTGCGGAATG | 667 |
|  |  | LJ-2-DR | TGGGGTGACGATGTGGGAGA |  |
|  | LJMV1 RNA3 | LJ-3-DF | ACCCCATTCCAATCTGCC | 758 |
|  |  | LJ-3-DR | ACCCCTCCTTAACGACCA |  |
|  | LJMV1 RNA4 | LJ-4-DF | AGGCGAAGTTGAAGGAGT | 650 |
|  |  | LJ-4-DR | CAGAATTGTGGGGAGAGTGA |  |
|  | LJMV1 RNA5 | LJ-5-DF | ACCAACCATTCTCTCCACAC | 679 |
|  |  | LJ-5-DR | CTTCTTCCTGCCATTGACCT |  |
|  | CJVV1 RNA1 | CJ-1-DF | ACAAAGTCTCCCGCCTCAAC | 513 |
|  |  | CJ-1-DR | GAACCTCACGGTCACCCATT |  |
|  | CJVV1 RNA2 | CJ-2-DF | CGGCGTTCGCATAGTAAGGT | 400 |
|  |  | CJ-2-DR | GTCGGCAATCGGGTTGTCTC |  |
|  | CJVV1 RNA3 | CJ-3-DF | AGAGGGTTGTGGTAGCATTG | 624 |
|  |  | CJ-3-DR | TGGTAACAGCGTATTCGGTA |  |
|  | CJVV1 RNA4 | CJ-4-DF | AGGTTCAGCGTTAGTTCGTT | 309 |
|  |  | CJ-4-DR | GTATTAGACCCGCAAAGTGG |  |
|  | CJVV1 RNA5a | CJ-5a-DF | CAGCAGTCGGATTGATAACC | 453 |
|  |  | CJ-5a-DR | ATCTGGAACCCTCTTGACCC |  |
|  | CJVV1 RNA5b | CJ-5b-DF | GAGTAAGCGGGTCAAGAGGG | 433 |
|  |  | CJ-5b-DR | CTGCGAGTTCTGACAATGCT |  |
