# Supplementary figures and images for: Conserved untranslated regions of multipartite viruses: Natural markers of novel viral genomic components and tags of viral evolution
Source: Virus Evol. 2024 Jan 12;10(1):veae004. doi: 10.1093/ve/veae004 (PMC10868557; doi:10.1093/ve/veae004)

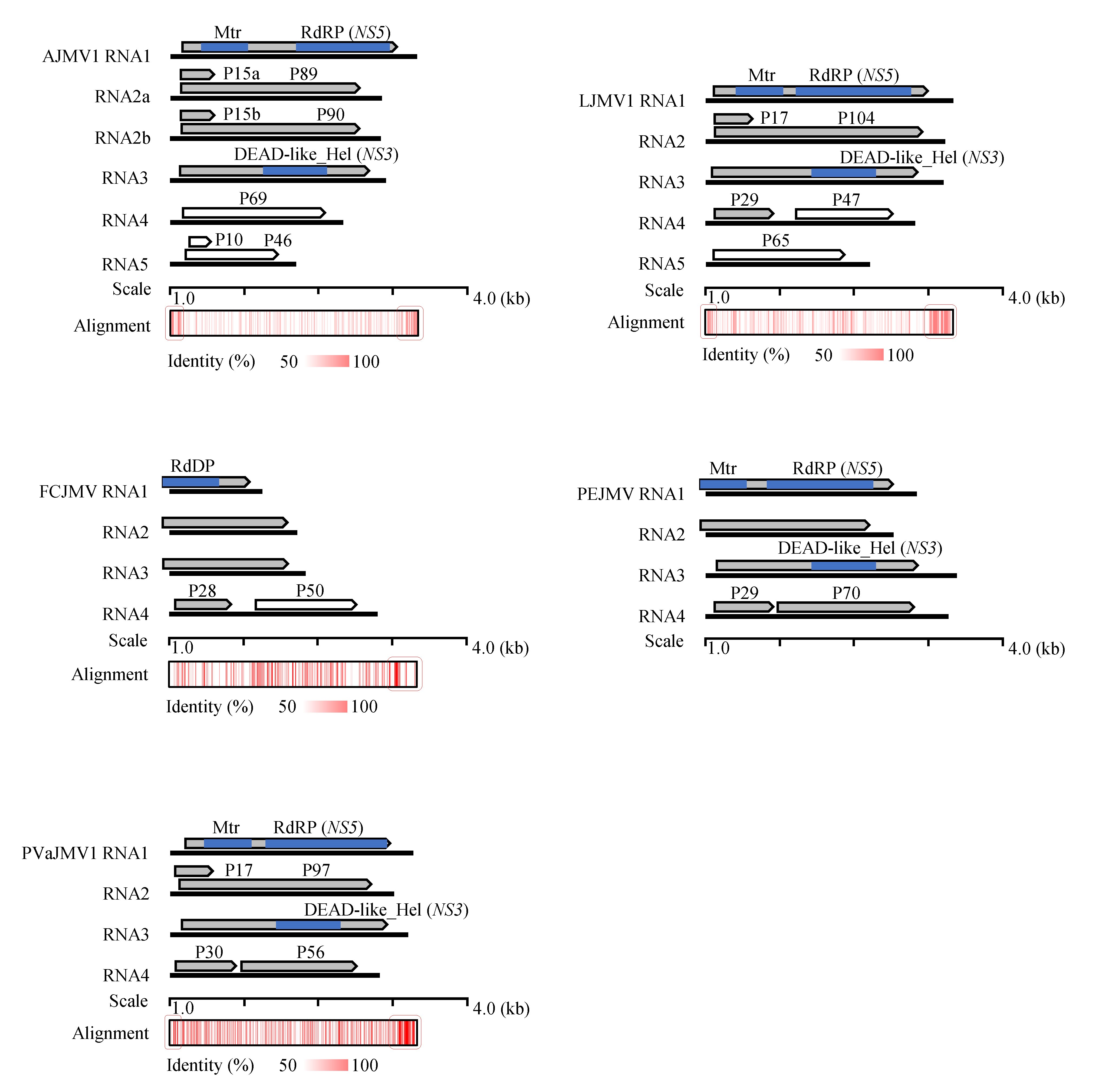

Supplement: veae004_Supp [file veae004_supp.zip › suppl_data/Supplementary Figure S1.jpg]

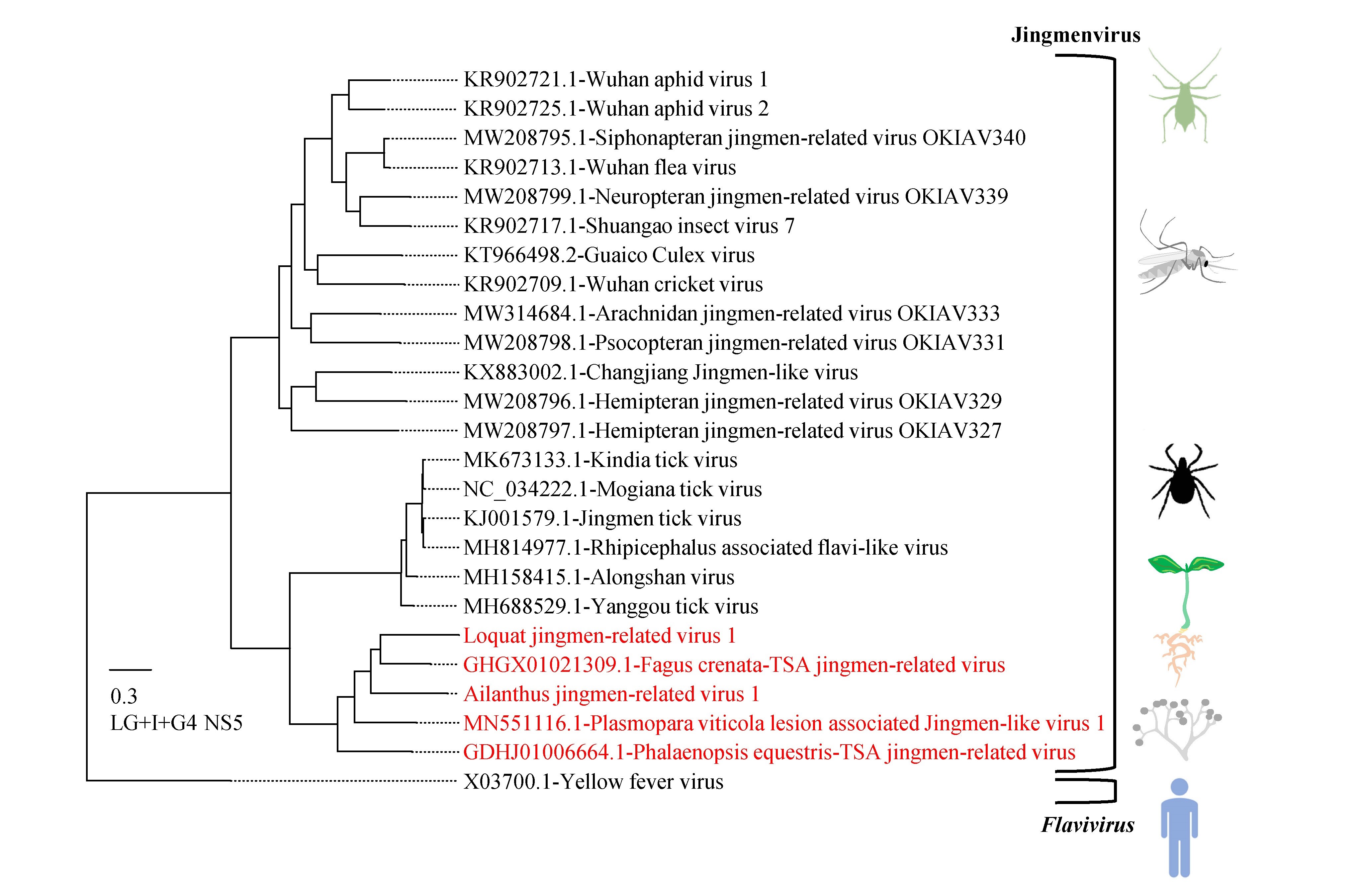

Supplement: veae004_Supp [file veae004_supp.zip › suppl_data/Supplementary Figure S2.jpg]

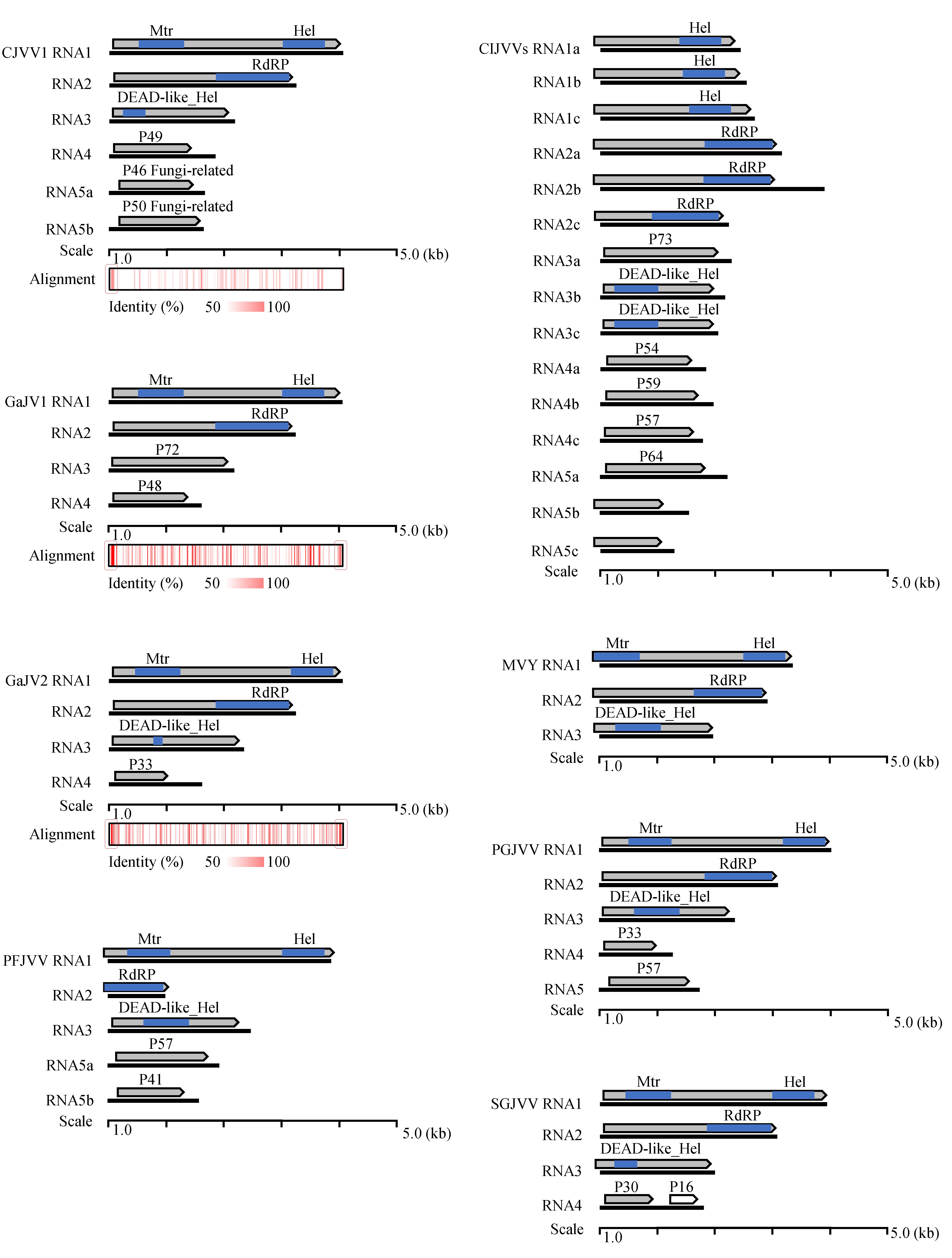

Supplement: veae004_Supp [file veae004_supp.zip › suppl_data/Supplementary Figure S3.jpg]

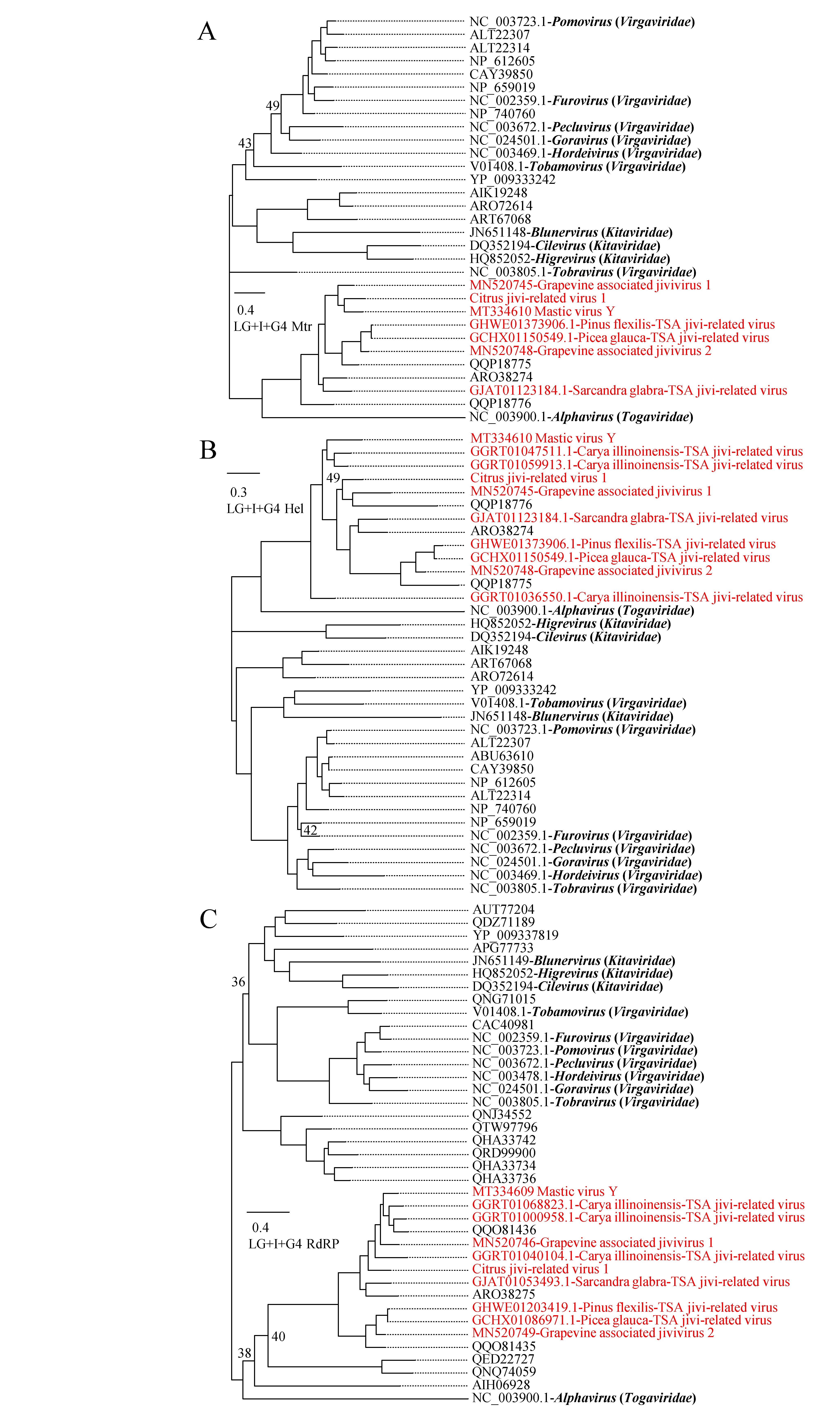

Supplement: veae004_Supp [file veae004_supp.zip › suppl_data/Supplementary Figure S4.jpg]

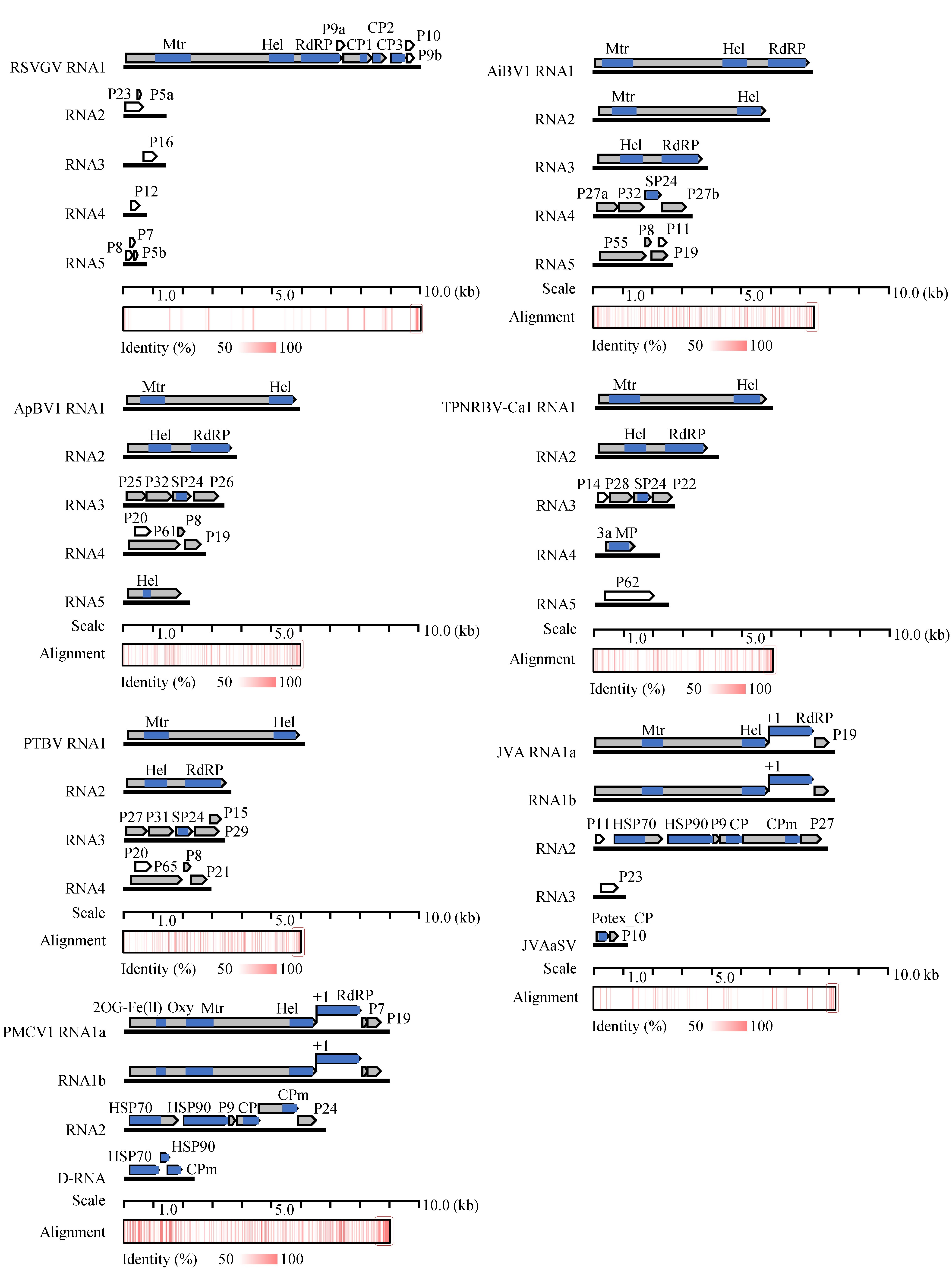

Supplement: veae004_Supp [file veae004_supp.zip › suppl_data/Supplementary Figure S5.jpg]

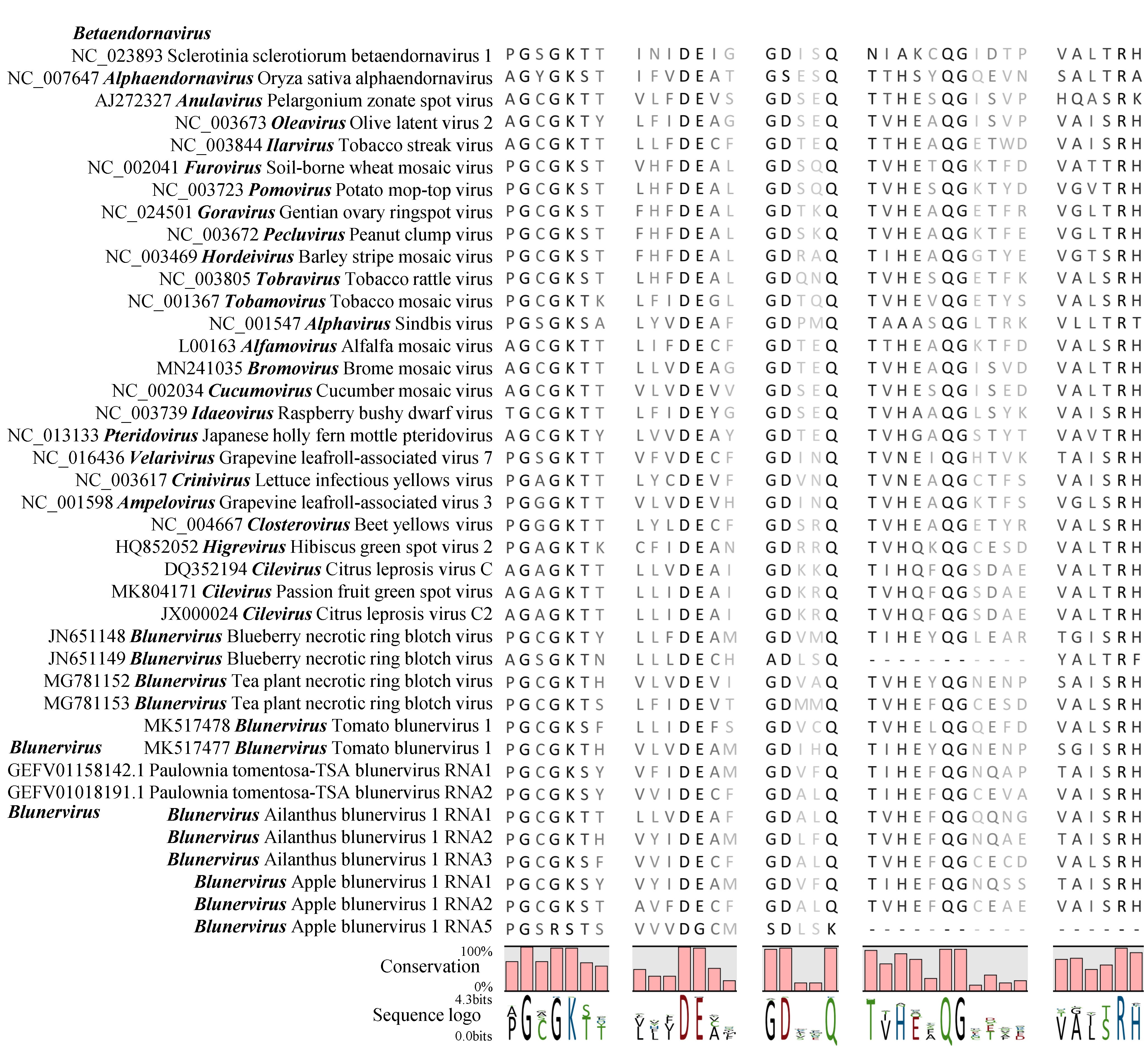

Supplement: veae004_Supp [file veae004_supp.zip › suppl_data/Supplementary Figure S6.jpg]
